# Steric Effects on the Photovoltaic Performance of Panchromatic Ruthenium Sensitizers for Dye-Sensitized Solar Cells

**DOI:** 10.1021/acsami.3c19298

**Published:** 2024-03-04

**Authors:** Chia-Yuan Chen, Ting-Yi Lin, Chi-Feng Chiu, Mandy M. Lee, Wei-Long Li, Min-Yu Chen, Tzu-Hao Hung, Zhao-Jie Zhang, Hui-Hsu Gavin Tsai, Shih-Sheng Sun, Chun-Guey Wu

**Affiliations:** † Research Center of New Generation Light Driven Photovoltaic Modules and, National Central University, Taoyuan 32001, Taiwan, R.O.C; ‡ Department of Chemistry, National Central University, Taoyuan 32001, Taiwan, R.O.C; § Institute of Chemistry, Academia Sinica, No. 128, Academia Road, Sec. 2, Nankang, Taipei 115, Taiwan, R.O.C

**Keywords:** dye-sensitized solar cells, heteroleptic ruthenium complexes, stereoisomer, panchromatic response, coadsorbent-free, steric effects

## Abstract

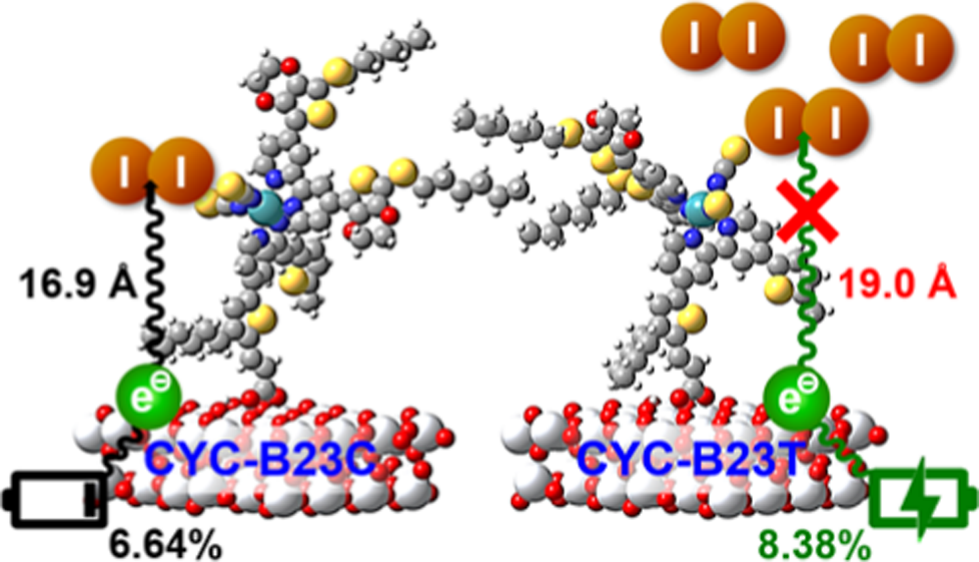

Three new heteroleptic Ru complexes, **CYC-B22**, **CYC-B23C**, and **CYC-B23T**, were prepared as sensitizers for coadsorbent-free, panchromatic, and efficient dye-sensitized solar cells. They are simultaneously functionalized with highly conjugated anchoring and ancillary ligands to explore the electronic and steric effects on their photovoltaic characteristics. The coadsorbent-free device based on **CYC-B22** achieved the best power conversion efficiency (PCE) of 8.63% and a panchromatic response extending to 850 nm. The two stereoisomers, **CYC-B23C** and **CYC-B23T** coordinated with an unsymmetrical anchoring ligand, display similar absorption properties and the same driving forces for electron injection as well as dye regeneration. Nevertheless, the devices show not only the remarkably distinct PCE (6.64% vs 8.38%) but also discernible stability. The molecular simulation for the two stereoisomers adsorbed on TiO_2_ clarifies the distinguishable distances (16.9 Å vs 19.0 Å) between the sulfur atoms in the NCS ligands and the surface of the TiO_2_, dominating the charge recombination dynamics and iodine binding and therefore the PCE and stability of the devices. This study on the steric effects caused by the highly conjugated and unsymmetrical anchoring ligand on the adsorption geometry and photovoltaic performance of the dyes paves a new way for advancing the molecular design of polypyridyl metal complex sensitizers.

## 1 Introduction

Dye-sensitized solar cells (DSC) stand out as an attractive photovoltaic (PV) technology suitable for diverse applications, both outdoors and indoors.^1−4^ In the devices, dye molecules adsorbed on mesoporous TiO_2_ act as the key light-harvesting component, surface passivation material, and photon-to-current conversion center. Therefore, the innovation to enhance power conversion efficiency (PCE) of the devices has been concentrated on molecular design of the dyes.^1,2,5−9^ The cells based on metal-free organic dyes and zinc porphyrins demonstrated a PCE beyond 13%,^10−14^ whereas the devices sensitized with polypyridyl ruthenium (Ru) complexes also reached the best PCE of ca. 12%.^15,16^ The comparable PV performance of the devices shows that the Ru complexes are good candidates for applications in DSCs, which is ascribed to the fact that their metal-to-ligand charge transfer (MLCT) and ligand-to-ligand charge transfer (LLCT) transitions are capable of covering the whole visible light. Furthermore, the suitable energy levels and proper localization of the frontier molecular orbitals, reversible redox behavior, and long-lived excited states favor the heterogeneous charge separation when applied in DSCs.^17^ These attractive features lead the devices to display a panchromatic response,^17−27^ even in the absence of cosensitization, which used dual (or multiple) dyes.^11−14,28^

In the molecular design of polypyridyl Ru complexes, decoration with conjugated moieties and attachment of alkyl pendants in the ligands were the popular strategies to red shift and enhance the MLCT transitions of the Ru complexes,^17,20,22−27^ prevent from dye aggregation, and suppress charge recombination (CR)^29^. In contrast to the branch of terpyridyl (tpy) tridentate ligands,^17,18,20,22−27^ several Ru complexes with bipyridyl (bpy) ancillary ligands bearing conjugated moieties and alkyl groups lead to a near-panchromatic response, high PCE, and good stability of the corresponding devices.^1,2,15,16,30−36^ Therefore, the impressive performance of the Ru complexes can be mainly ascribed to the multifunction of the bpy-based ancillary ligands. Beyond 4,4′-dicarboxy-2,2′-bipyridine (dcbpy), the most common anchoring ligand, the insertion of alkyl-substituted conjugation moieties in the bpy-based anchoring ligands (between bipyridine and carboxylic acids) is challenging because of the amphiphilic nature of the ligands requiring sophisticated synthesis procedures. Consequently, there has been a scarcity of literature on Ru complexes with extensively conjugated and symmetrical bpy-based anchoring ligands.^37−42^ These works demonstrated that the conjugated moieties in the anchoring ligands can stabilize the potential of excited states of the Ru complexes, thereby red-shifting the MLCT and LLCT transitions. Nevertheless, there is a notable gap in our understanding regarding the profound effects of highly conjugated anchoring ligands on the adsorption geometry of Ru complexes on TiO_2_, their interaction with iodine, and the resulting charge transfer dynamics in these devices. Furthermore, there are no reports yet on Ru complexes coordinated with unsymmetrical anchoring ligands and their stereoisomeric effects. On the other hand, coadsorbents [such as chenodeoxycholic acid (CDCA)] have the potential to substantially enhance both the PCE and durability of the devices.^43,44^ Their success could be ascribed to the prevention of dye aggregation and passivation of the TiO_2_ surface. However, the ratio between dyes and the coadsorbents in the dye solutions must be fine-tuned, and the actual proportion of the two materials will deviate from the optimal condition after use, which are unfavorable to the mass production of the devices. Hence, the development of dye molecules showing controllable steric effects for a coadsorbent-free system is valuable to the simplification and economization of DSC fabrication.^45,46^

**Figure 1:**
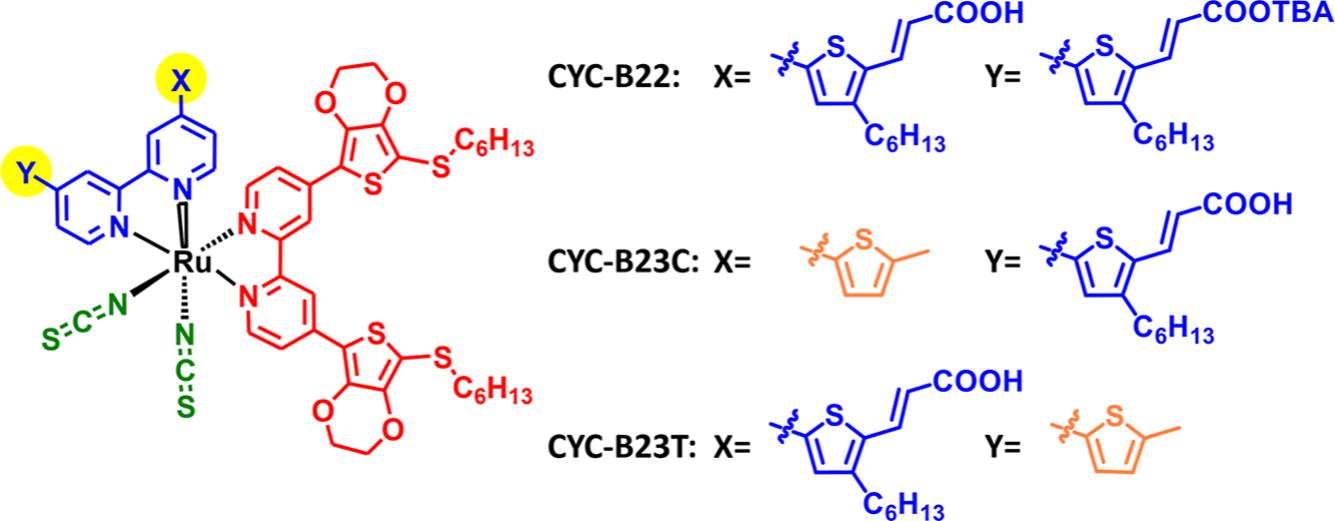
Molecular structures of three new heteroleptic Ru complexes (**CYC-B22**, **CYC-B23C**, and **CYC-23T**, wherein TBA is tetrabutylammonium).

In this study, three new heteroleptic Ru complexes (named **CYC-B22**, **CYC-B23C**, and **CYC-B23T**) are designed as dyes for DSC applications. As presented in Figure 1, all three Ru complexes have highly conjugated anchoring and ancillary ligands, both bearing thienylenes for the first time. In addition to 2-thiohexyl-3,4-ethylenedioxy-thiophene (TH-EDOT) applied in their ancillary ligands, the anchoring ligand of **CYC-B22** has extended conjugation by inserting hexyl-thienylene-vinylene moieties between the bpy unit and carboxylic acids. Additionally, hexyl groups are attached to both of the ligands for controlling the steric effects on suppressing dye aggregation and CR in the devices. Compared with the homoleptic forms using two dcbpy anchoring ligands (such as **N3** and **N719**), which can densely adsorb on TiO_2_ and lead the devices to show high open-circuit voltage (*V*_oc_),^1^ the heteroleptic Ru complexes functionalized with conjugated units usually have larger molecular volumes, less dye-loadings, and lower cell *V*_oc_ despite the higher short-circuit current density (*J*_sc_) of the devices.^2^ Therefore, alkyl groups in the anchoring ligand may shield the dye-free TiO_2_ surface from CR and improve the *V*_oc_ for the devices. In two stereoisomers (**CYC-B23C** and **CYC-B23T**), one of the carboxylated hexyl-thienylene-vinylene units in the symmetrical anchoring ligand for **CYC-B22** is replaced with 2-methylthiophene. As far as we know, this is the first report comprehensively studying the steric effects induced by highly conjugated and unsymmetrical anchoring ligands of two stereoisomers on device characteristics. This study aims to advance the molecular design of heteroleptic metal complex sensitizers for coadsorbent-free and panchromatic DSC. For highlighting the features of the three new heteroleptic Ru complexes, the lowest energy MLCT transitions and device characteristics of other Ru complexes coordinated with conjugation-extended bpy anchoring ligands are summarized in Table S1. Among them, **CYC-B22** displays the best light-harvesting capacity for coadsorbent-free, panchromatic, and efficient DSC. Furthermore, **CYC-B23C** and **CYC-B23T** demonstrate for the first time that stereoisomerism significantly affects the PV parameters of the devices, despite their similar absorption properties. These new findings in this study suggest that employing highly conjugated and unsymmetrical anchoring ligands in metal complexes shows promise in balancing the maximization of light harvesting with the minimization of CR.

## 2 Experimental Section

### 2.1 Materials and Measurements

The chemicals for synthesizing the ligands and the Ru complex as well as fabricating devices were acquired from commercial sources and utilized as received unless otherwise specified. The structural characterization of the intermediates, esterified anchoring ligands (L22-ester and L23-ester), and three new Ru complexes (**CYC-B22**, **CYC-B23C**, and **CYC-B23T**) was done using proton nuclear magnetic resonance (^1^H NMR) spectroscopy. The molecular structures of the final ruthenium complexes were also identified with Fourier-transform infrared spectrometry (FTIR), high-resolution fast atom bombardment mass spectrometry (HRFAB-MS), and elemental analysis. The ^1^H NMR spectra were measured in deuterated chloroform (CDCl_3_) or dimethyl sulfoxide (DMSO-*d*_6_) using a 300 (or 500) MHz NMR spectrometer (Brüker). The FTIR spectra of the three complexes were obtained in the form of KBr pellets by using a FTIR spectrometer (FT/IR-4100, Jasco). The HRFAB-MS spectra were measured with an HRMS instrument (JMS-700, JEOL). Elemental analysis was performed with an elemental analyzer (CHNOS Rapid-F002 analysis system, Heraeus). The melting points (mp) were measured using a specific apparatus (MP-2D, Fargo). Absorption spectra and electrochemical properties of the new Ru complexes were studied using the same equipment and conditions as we previously reported.^17^

### 2.2 Synthesis of Esterified Anchoring Ligands (L22-Ester and L23-Ester)

**Figure 2:**
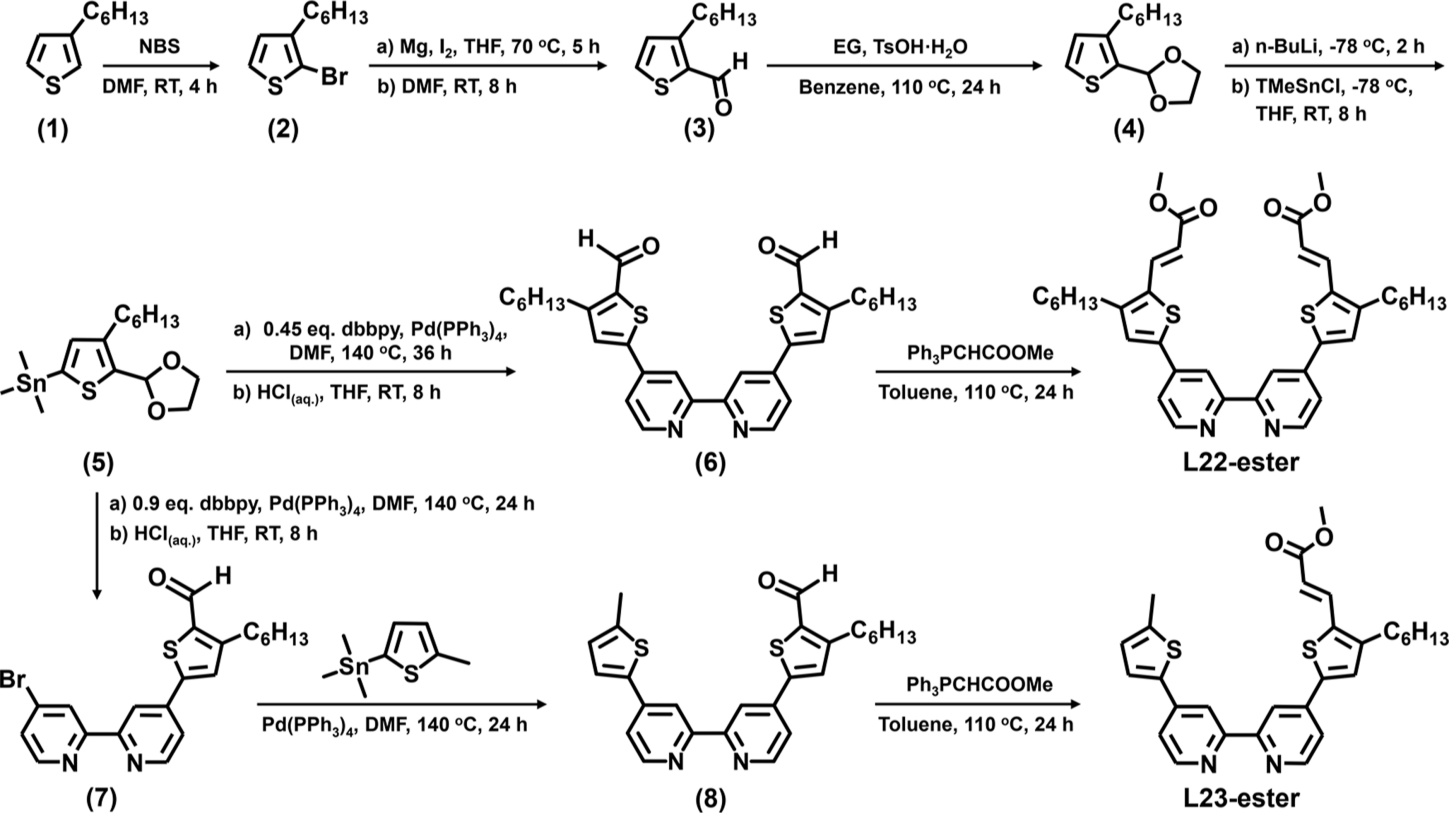
Synthetic scheme of esterified anchoring ligands (L22-ester and L23-ester).

The synthetic scheme of the two esterified anchoring ligands (L22-ester and L23-ester) is displayed in Figure 2. The details are available in the Supporting Information.

### 2.3 Synthesis of CYC-B22 [Ru(L22)(L20)(NCS)_2_]

The heteroleptic Ru complex was prepared with the one-pot reaction protocol similar to our previous report.^42^ 0.337 g (0.55 mmol) of [RuCl_2_(*p*-cymene)]_2_ and 0.736 g (1.10 mmol) of ancillary ligand bearing two 2-thiohexyl-3,4-ethylenedioxy-thiophene (TH-EDOT) units (L20) were prepared according to the literature;^47^ 0.692 g (1.10 mmol) of L22-ester and excess ammonium thiocyanate (NH_4_NCS) were added in a Schlenk flask containing DMF and then hydrolyzed with 0.115 g (2.75 mmol) LiOH·H_2_O in water. After refluxing for 12 h, dilute HNO_3(aq.)_ was used to obtain precipitates. The collected crude products were washed with water, dried under vacuum, and then dissolved in a mixture of methanol and tetrabutylammonium hydroxide (TBAOH). The purification was carried out by using a Sephadex LH-20 column with methanol as an eluent. After collecting the main band, dilute HNO_3(aq.)_ was employed to lower the pH value to ca. 5.7. The filtered solids were washed using water and then dried under a vacuum. Upon repeating the purification for three times, 0.170 g (0.097 mmol, 35.1% yield) of **CYC-B22** was obtained. ^1^H NMR (500 MHz, δ/ppm in DMSO-*d*_6_): 9.16 (d, *J* = 6.0 Hz, 1H), 9.15 (d, *J* = 6.0 Hz, 1H), 8.91 (s, 1H), 8.74 (s, 1H), 8.67 (s, 1H), 8.51 (s, 1H), 8.09 (s, 1H), 8.00 (s, 2H), 7.84 (s, 1H), 7.63 (d, *J* = 15.0 Hz,1H), 7.50 (s, 2H), 7.48 (d, *J* = 15.0 Hz,1H), 7.30 (s, 2H), 6.38 (d, *J* = 14.5 Hz, 1H), 6.23 (d, *J* = 14.5 Hz,1H), 4.42 (q, 8H), 3.16 (t, *J* = 8.3 Hz, 8H), 2.87 (t, *J* = 6.6 Hz, 2H), 2.74 (m, 4H), 2.61 (m, 2H), 1.58 (m, 16H), 1.29 (m, 32H), 0.94 (t, *J* = 7.3 Hz, 12H), 0.86 (m, 6H), 0.79 (m, 6H). FTIR (KBr pellet, cm^–1^): 3041 (=C–H), 2956 (C–H), 2924 (C–H), 2098 (N=C=S), 1700 (C=O), 1608 (C=C), 1491 (C–H), 1361 (C–H), 1083 (C–O). MS: calcd. *m*/*z* 1756.56 ([M]^+^); HRFAB-MS found, 1514.2854 ([M-N(C_4_H_9_)_4_]^+^). Elemental anal. calcd for C_88_H_115_N_7_O_8_RuS_8_·2H_2_O: C, 58.96; H, 6.69; N, 5.47%. Found: C, 58.97; H, 6.61; N, 5.52%. mp.: 235 °C.

### 2.4 Synthesis of CYC-B23C and CYC-B23T [Ru(L23)(L20)(NCS)_2_]

The two stereoisomers were also synthesized by the same procedure as that for **CYC-B22**, except that the unsymmetrical ligand (L23-ester) was used in place of L22-ester. For the two stereoisomers, 0.341 g (0.56 mmol) of [RuCl_2_(*p*-cymene)]_2_, 0.745 g (1.11 mmol) of L20, 0.560 g (1.11 mmol) of L23-ester, and excess NH_4_NCS were used in the reaction. The two stereoisomers were separated using recrystallization with acetone and then silica gel chromatography, successively using dichloromethane/methanol = 20/1 and hexane/dichloromethane/acetone/methanol = 50/50/10/1 as the eluents. The stereoisomers were individually dissolved in tetrahydrofuran (THF) and hydrolyzed with LiOH·H_2_O in water. After refluxing for 8 h, dilute HNO_3(aq.)_ was added into the solutions to get precipitates. The crude products were collected, washed using water, dried under vacuum, and then dissolved in a mixture of methanol and TBAOH for purification with a Sephadex LH-20 column, successively using methanol and methanol/dichloromethane = 3/2 as the eluents. The pH value of the collected main band was lowered to ca. 1 through adding dilute HNO_3(aq.)_. The collected solids were washed using water and then dried under a vacuum. Upon purification, 0.062 g (0.045 mmol, 16.2% yield) and 0.069 g (0.050 mmol, 18.0% yield) of **CYC-B23C** and **CYC-B23T** were obtained, respectively.

#### 2.4.1 CYC-B23C

^1^H NMR (500 MHz, δ/ppm in DMSO-*d*_6_): 9.23 (d, *J* = 5.7 Hz, 1H), 9.17 (d, *J* = 5.7 Hz, 1H), 9.01 (s, 1H), 8.86 (s, 1H), 8.72 (s, 1H), 8.54 (s, 1H), 8.17 (d, *J* = 5.0 Hz, 1H), 8.13 (s, 1H), 8.09 (d, *J* = 5.5 Hz, 1H), 7.92 (d, *J* = 3.0 Hz, 1H), 7.82 (d, *J* = 15.5 Hz, 1H), 7.57 (d, *J* = 5.8 Hz, 1H), 7.53 (d, *J* = 5.8 Hz, 1H), 7.38 (d, *J* = 5.8 Hz, 1H), 7.33 (d, *J* = 5.8 Hz, 1H), 7.00 (s, 1H), 6.28 (d, *J* = 15.5 Hz, 1H), 4.43 (m, 8H), 2.87 (t, *J* = 7.2 Hz, 2H), 2.79 (m, 4H), 2.62 (s, 3H), 1.68 (m, 2H), 1.61 (m, 2H), 1.51 (m, 2H), 1.30 (m, 18H), 0.81 (m, 9H). FTIR (KBr pellet, cm^–1^): 3052 (=C–H), 2921 (C–H), 2889 (C–H), 2100 (N=C=S), 1701 (C=O), 1608 (C=C), 1489 (C–H), 1361 (C–H), 1086 (C–O). MS: calcd 1374.20 ([M]^+^); HRFAB-MS found, 1374.2015 ([M]^+^). Elemental anal. calcd for C_64_H_68_N_6_O_6_RuS_8_·2H_2_O: C, 54.48; H, 5.14; N, 5.96%. Found: C, 54.67; H, 5.14; N, 5.97%. mp.: 249 °C.

#### 2.4.2 CYC-B23T

^1^H NMR (500 MHz, δ/ppm in DMSO-*d*_6_): 9.16 (d, *J* = 3.0 Hz, 1H), 9.15 (d, *J* = 3.0 Hz, 1H), 9.01 (s, 1H), 8.89 (s, 1H), 8.73 (s, 1H), 8.56 (s, 1H), 8.10 (s, 2H), 8.08 (d, *J* = 5.8 Hz, 1H), 7.92 (s, 1H), 7.69 (d, *J* = 15.5 Hz, 1H), 7.62 (d, *J* = 6.1 Hz, 1H), 7.58 (*J* = 6.1 Hz, 1H), 7.41 (d, *J* = 5.6 Hz, 2H), 7.11 (s, 1H), 6.53 (s, 1H), 6.16 (d, *J* = 15.5 Hz, 1H), 4.43 (m, 8H), 2.88 (t, *J* = 7.2 Hz, 2H), 2.79 (t, *J* = 7.2 Hz, 2H), 2.72 (t, *J* = 7.0 Hz, 2H), 2.62 (s, 3H), 1.60 (m, 4H), 1.52 (m, 2H), 1.42 (m, 2H), 1.28 (m, 16H), 0.83 (m, 9H). FTIR (KBr pellet, cm^–1^): 3051 (=C–H), 2928 (C–H), 2851 (C–H), 2099 (N=C=S), 1700 (C=O), 1608 (C=C), 1490 (C–H), 1361 (C–H), 1086 (C–O). MS: calcd 1374.20 ([M]^+^); HRFAB-MS found, 1374.2020 ([M]^+^). Elemental anal. calcd for C_64_H_68_N_6_O_6_RuS_8_·2H_2_O: C, 54.48; H, 5.14; N, 5.96%. Found: C, 54.71; H, 5.06; N, 5.98%. mp.: 251 °C.

### 2.5 Theoretical Calculation

Density functional theory (DFT) computation based on the Gaussian 09 program^48^ was used to investigate the geometry and electronic transitions of the three new Ru complexes, in which the exchange–correlation functional, the basis set, and the model for solvent effect used for the ground state optimization were the same as those in our previous report.^17^ All hexyl groups in the Ru complexes were substituted with methyl moieties to alleviate the computational costs. To mimic the partial deprotonation of the complexes dissolved in DMF, monodeprotonated Ru complexes were investigated. Furthermore, time-dependent (TD) DFT calculations were performed under the same level and model^17^ for the three new complexes. The geometry optimization of the complexes adsorbed onto a (TiO_2_)_64_ unit cell, employing periodic boundary conditions, was conducted using the DFT method within the DMol3 program.^49^ The DFT calculations were based on the Generalized Gradient Approximation and the Perdew–Burke–Ernzerhof (PBE) exchange–correlation functional^50^ along with the double-numerical with polarization atomics (DNP) basis set. It is noteworthy that an all-electron numerical method was utilized.^51^

### 2.6 Device Fabrication

The fluorine-doped tin oxide (FTO)-coated glass was cleaned, followed by a pretreatment using TiCl_4(aq.)_.^17^ A 4 μm-thick transparent TiO_2_ layer based on the 18NR-T paste (GreatCell Solar) was printed and calcined at 500 °C for 1 h. A 6 μm thick scattering layer using TiO_2_ beads^52^ and a 5 μm thick scattering layer using WER2-O TiO_2_ paste (GreatCell Solar) were successively overlaid on the transparent layer. After post-treatment,^17^ the electrode was then immersed in the dye solution (0.1 mM) at −18 °C for 30 h, using a mixed solvent of acetonitrile, DMSO, and *t*-butanol (volume ratio = 1:1:1). A solution containing 0.6 M 1-butyl-3-methylimidazolium iodide (BMII), 0.1 M LiI, 0.03 M I_2_, 0.5 M 4-*tert*-butylpyridine (*t*BP), and 0.1 M guanidinium thiocyanate (GuNCS) in acetonitrile was used as the electrolyte. For stability tests under a storage temperature of 26 ± 4 °C and relative humidity of 75 ± 10% as well as the thermally accelerated aging at 70 ± 3 °C, another electrolyte consisting of 1.0 M BMII, 0.15 M I_2_, 0.1 M GuNCS, and 0.5 M *N*-butylbenzimidazole (NBB) in 3-methoxypropionitrile (MPN) was employed.

### 2.7 Device Characterization

An anodized aluminum mask (aperture area of 0.12 cm^2^) was mounted on the top of the devices for the designated area and to minimize unwanted light reflection and diffusion.^53^ A source measure unit (Keithley 2400) and a sampling delay time of 1 s (equal to the scan rate of 8.8 mV s^–1^) were used for the measurement of the photocurrent density–voltage (*J*–*V*) curves of the devices under the standard testing conditions (STC; AM 1.5 global spectrum and cell temperature of 25 °C).^17,53^ An AAA-class solar simulator (YCSS-50, Yamashita Denso Co.) was used as the light source, having a total irradiance of 100 mW cm^–2^ adjusted with a KG2-equipped single-crystalline silicon PV reference cell (cSi-RC). For accuracy and traceability to the National Institute of Advanced Industrial Science and Technology, Japan, the cSi-RC was calibrated (secondary). The spectral irradiance of the light source was obtained using a spectroradiometer (S-2440 model II, SOMA Optics Ltd.). The temperature of the devices under test was 25 ± 1 °C, monitored and controlled with a T-type thermocouple and a water-cooling sample stage, respectively. The incident photon-to-current efficiency (IPCE) spectra of the devices were measured using the same IPCE measurement system and a direct current mode^54^ as we reported.^17^ The transient absorption spectra (TAS) were measured using the same system,^17^ except that a 12 μm thick transparent TiO_2_ layer and the optimized electrolyte based on acetonitrile as solvent (provided above) were employed for the coadsorbent-free devices. Moreover, the decay of the absorption was probed at 920 nm. Intensity-modulated photovoltage/photocurrent spectroscopy (IMVS/IMPS)^17^ and the charge extraction technique^55,56^ with a warm white LED lighting source were employed to investigate the dynamics of charge transfer in the devices.

## 3 Results and Discussion

### 3.1 Synthesis of New Ru Complexes

**Figure 3:**
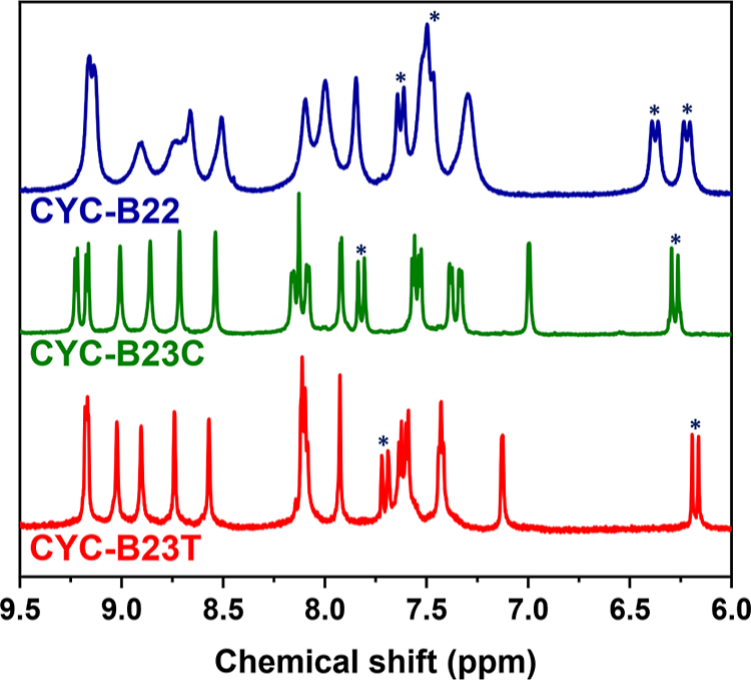
Proton NMR spectra (aromatic region) of **CYC-B22**, **CYC-B23C**, and **CYC-B23T** measured in DMSO-*d*_6_ (wherein signals labeled with asterisks represent vinyl protons).

For covalent immobilization of Ru complexes on the TiO_2_ and rapid electron transfer from the photoexcited Ru complexes to the TiO_2_, the anchoring ligand bearing two carboxylic acids (such as dcbpy) was regularly used.^1,2^ However, carboxylic acids confer hydrophilicity to the highly conjugated and hydrophobic backbone of the ligands, which unfortunately increases the difficulty in the synthesis and purification of the ligands as well as the final heteroleptic Ru complexes. To overcome the dilemma, esterified anchoring ligands were strategically used for the preparation of the three new Ru complexes; thereafter, hydrolysis was performed to obtain carboxylic acid groups. The signals of aromatic protons in the NMR spectra of the new Ru complexes (**CYC-B22**, **CYC-B23C**, and **CYC-B23T**) measured in DMSO-*d*_6_ are presented in Figure 3. The multiple signals in the aromatic region indicate that all three Ru complexes are in a cis configuration. The doublet signals labeled with asterisks show coupling constants (*J*) in the range of 14.5–15.5 Hz, confirming all of the vinyl groups inserted between carboxylic acids and hexylthiophenes for the three Ru complexes are in an E-form. The E-form dyes will be beneficial to the electron transfer from the excited states of the Ru complexes to the conduction band of TiO_2_,^7^ thereby enhancing the photocurrent density of the devices. Moreover, the vinyl protons in the anchoring ligands of **CYC-B23C** and **CYC-B23T** are excellent indicators to distinguish the two stereoisomers. Compared with **CYC-B23C**, the upfield shift of the vinyl protons in **CYC-B23T** is due to a more effective shielding effect induced by the anionic NCS ligand quasi-linearly positioned to the vinyl group. It is also noted that the ratios of **CYC-B23C** to **CYC-B23T** estimated from the proton NMR spectrum of the crude product (ester forms) and the finally isolated yields (acid forms) are around 50%/50% and 16.2%/18.0%, respectively. These data suggest that the two stereoisomers are thermodynamically identical in the synthesis. In other words, the steric effects prompted by the unsymmetrical anchoring ligand do not favor any specific orientation for the coordination.

### 3.2 Absorption and Electrochemical Properties of Ru Complexes

**Figure 4:**
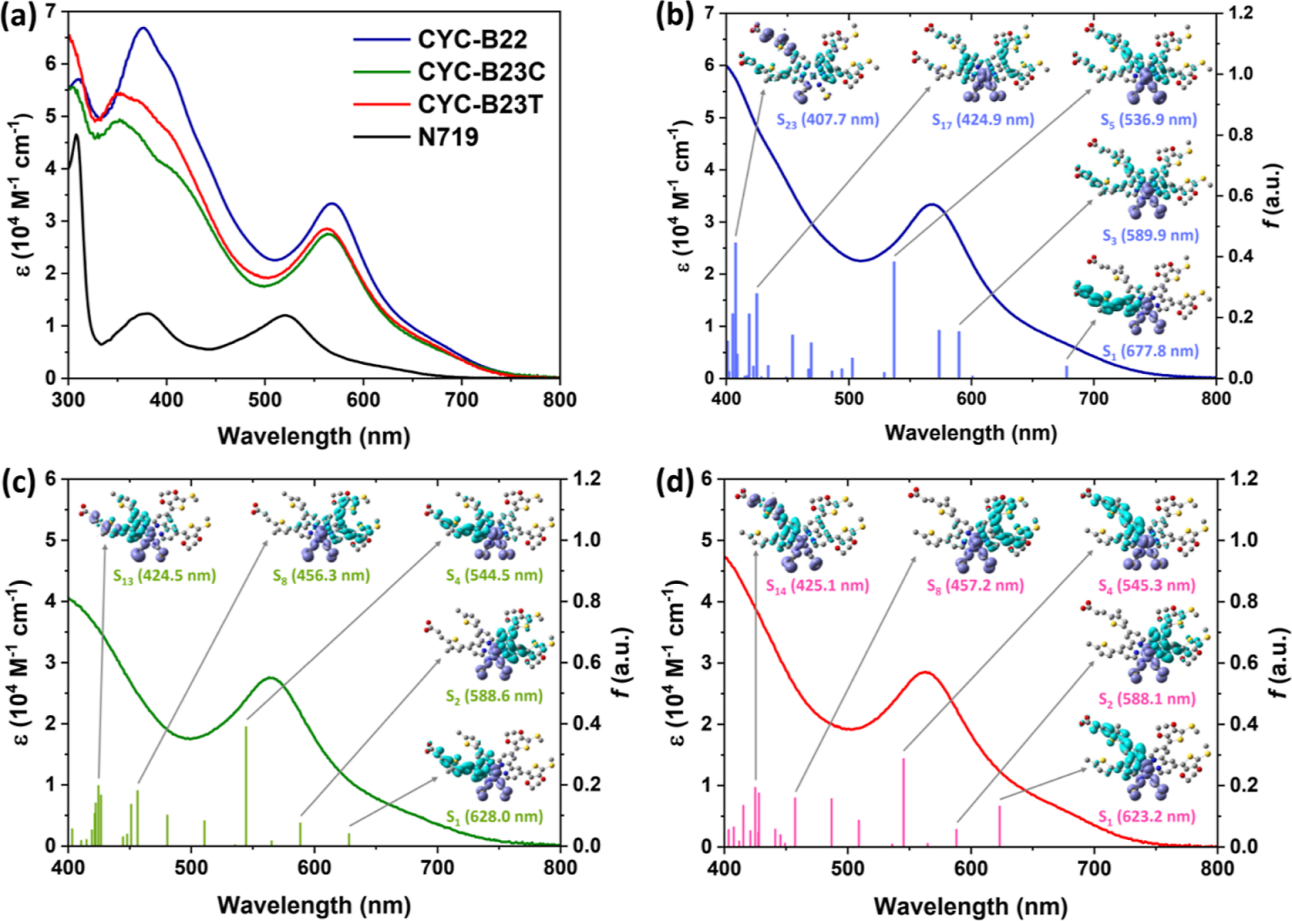
(a) Absorption spectra of three new Ru complexes and **N719** measured in DMF. Vertical transitions and associated EDDMs obtained by using the TDDFT method for (b) **CYC-B22**, (c) **CYC-B23C**, and (d) **CYC-B23T**. In the EDDMs, protons are excluded for clarity, and the electrons (cyan) and holes (purple) are displayed with an isovalue of 0.001 e Å^–3^.

Figure 4a shows the absorption spectra of the three new Ru complexes and **N719** in DMF. The wavelength of maximum absorbance (λ_max_) and the corresponding molar absorption coefficient (ε) are summarized in Table 1. **CYC-B22**, **CYC-B23C**, and **CYC-B23T** all exhibit three prominent absorption bands within the wavelength range of 300–800 nm. It is noteworthy that **CYC-B22** exhibits the lowest-energetic MLCT transition band at 569 nm and the highest ε = 3.34 × 10^4^ M^–1^ cm^–1^, which outperforms the other heteroleptic Ru sensitizers using conjugation-extended bpy anchoring ligands (Table S1). In contrast to those of **CYC-B23C** (563 nm with ε = 2.76 × 10^4^ M^–1^ cm^–1^) and **CYC-B23T** (563 nm with ε = 2.86 × 10^4^ M^–1^ cm^–1^), the bathochromic shift of 6 nm and the increased (>16%) absorbance of **CYC-B22** are attributed to the highly conjugated and symmetrical anchoring ligand. Compared with those of **N719** (521 nm with ε = 1.20 × 10^4^ M^–1^ cm^–1^), the superior light-harvesting characteristics of the three new Ru complexes indicate that the higher degree of conjugation in both the anchoring and ancillary ligands results not only in a reduced energy gap but also in an enhanced absorbance of the low-lying MLCT bands. It is noted here that **CYC-B23C** has an absorption profile similar to that of its stereoisomer (**CYC-B23T**), which suggests that the spatial difference of carboxylated hexyl-thienylene-vinylene and 2-methylthiophene with respect to the NCS ligands has negligible effects on the light-harvesting capability of the Ru complexes in DMF.

**Table 1:** Absorption Properties and Potentials of the Ru Complexes Measured in DMF

complex	λ_max_ [nm] (ε [10^4^ M^–1^ cm^–1^])		*E*_ox_^a^ (V vs NHE)	*E*_0–0_^b^ (eV)	E*^c^ (V vs NHE)
CYC-B22	377 (6.69)	569 (3.34)	0.88	1.52	–0.64
CYC-B23C	352 (4.94)	563 (2.76)	0.88	1.56	–0.68
CYC-B23T	353 (5.45)	563 (2.86)	0.88	1.56	–0.68
N719	379 (1.24)	521 (1.20)	1.08	1.70	–0.62

a The oxidation potentials of the Ru complexes in their ground state were obtained from the first oxidation peaks observed in the square-wave voltammograms.

b The optical transition energy of each Ru complex in DMF was estimated using the absorption onset wavelength.

c The excited state potentials of the Ru complexes were obtained from *E*_ox_–E_0–0_.

TDDFT simulation was performed to gain a more profound comprehension of the transition behaviors of the three new Ru complexes in DMF. As shown in Figure 4b–d, the relative transition energy and oscillator strength (*f*) align well with the experimental observations, which reveal the best light-harvesting properties of **CYC-B22**. The electron density difference maps (EDDMs) embedded in Figure 4b for **CYC-B22** elucidate that the lowest-lying singlet transition at 677.8 nm (S_1_ and *f* = 0.0400) notably originates from the electron transfer from the mixed orbitals of the Ru center and NCS ligands to the anchoring ligand functionalized with two carboxylated hexylthienylene-vinylene (the MLCT transition advantages to the photocurrent density of the devices). The transitions at 589.9 nm (S_3_ and *f* = 0.1525), 536.9 nm (S_5_ and *f* = 0.3823), and 424.9 nm (S_17_ and *f* = 0.2778) should be partially applied to the favorable electron transfer process (from the excited states of **CYC-B22** to the conduction band of TiO_2_), due to the scenario that the highly conjugated anchoring and ancillary ligands are both involved in the delocalization of electrons. More importantly, the transitions associated with the anchoring ligand are thought to contribute positively to the photocurrent density of the devices.^17^ The transition at 407.7 nm (S_23_ and the highest *f* = 0.4448) includes both the intraligand π–π* transition and the charge transfers from the mixed orbitals of the Ru center and NCS ligands to the highly conjugated anchoring ligand, which is beneficial for the conversion of blue light into electricity for the devices. The localization of holes for the specified transitions of **CYC-B23C** (Figure 4c) is mainly dominated by Ru-NCS mixed orbitals. For the transitions at 628.0 nm (S_1_ and *f* = 0.0399), 544.5 nm (S_4_ and *f* = 0.3892), and 424.5 nm (S_13_ and *f* = 0.1967), the electrons locate in the anchoring ligand, in particular, bpy unit and carboxylated hexyl-thienylene-vinylene. These transitions could have a positive effect on the photocurrent density of the devices. However, it is found that the distributions of electrons for the transitions of **CYC-B23C** at 588.6 nm (S_2_ and *f* = 0.0744) and 456.3 nm (S_8_ and *f* = 0.1803) are both centralized in the ancillary ligand bearing two TH-EDOT moieties. These transitions should have limited contribution to the photocurrent density of the devices because the localization of electrons in the ancillary ligand is far away from TiO_2_, which hampers the electron injection. As presented in Figure 4d, the energy and strength (*f*) of the singlet transitions for **CYC-B23T** are similar to those of its stereoisomer, **CYC-B23C**, which is greatly consistent with the measured absorption spectra. Furthermore, it is noted that the electrons for the transitions of **CYC-B23T** at 623.2 nm (S_1_ and *f* = 0.132), 545.3 nm (S_4_ and *f* = 0.2871), and 425.1 nm (S_14_ and *f* = 0.1931) are mainly localized in the anchoring ligand. These features are comparable with those of **CYC-B23C**, although the orientation of carboxylated hexylthienylene-vinylene with respect to the NCS ligands is different. One may expect the charge transfer behaviors of the two stereoisomers will be similar when they are applied in DSC. However, it is found that the λ_max_ of three new Ru complexes adsorbed on TiO_2_ thin films is hypsochromically shifted, compared with those measured in DMF (Figure S1). These results should be due to the deprotonation of carboxylic acids for binding the Ru complexes on TiO_2_ and the different adsorption orientations of molecules. More importantly, it is noted that the absorbance of **CYC-B23C** on TiO_2_ is higher than that of **CYC-B23T**, suggesting that the adsorption behaviors of the two stereoisomers are influenced by the unsymmetrical anchoring ligand. Therefore, the steric effects of the two stereoisomers are expected to be crucial in influencing the PV parameters of the devices.

The energy levels of frontier molecular orbitals of **CYC-B22**, **CYC-B23C**, **CYC-B23T**, and **N719** are determined using square-wave voltammetry (Figure S2) and the optical transition energy (*E*_0–0_) obtained from the absorption onset wavelength (Table 1). The schematic energy diagram of the conduction band edge of anatase TiO_2_ (−0.50 V vs NHE), the three new Ru complexes, and the potential of the iodide/triiodide redox couple (0.35 V vs NHE) are displayed in Figure S3. These data indicate that the ground and excited states of the new Ru complexes potentially meet the crucial criteria for driving forces,^1^ promoting electron transfer from the excited states of Ru complexes to the conduction band of TiO_2_ and facilitating the regeneration of the complexes by the redox couple. In contrast to **N719**, TH-EDOT moieties integrated in the ancillary ligands of **CYC-B22**, **CYC-B23C**, and **CYC-B23T** significantly destabilize the potential (0.2 V) of the highest occupied molecular orbital (HOMO). In a comparison of **CYC-B23C** and **CYC-B23T**, **CYC-B22** shows the downwardly shifted potential of the lowest unoccupied molecular orbital (LUMO), due to the participation of one more carboxylate group in the anchoring ligand. It is also discovered that the HOMO and LUMO potentials of **CYC-B23C** and **CYC-B23T** are identical, which means the two stereoisomers applied in DSC have the same driving forces of transferring electrons to TiO_2_ and regenerating the complexes.

### 3.3 Photovoltaic Performance of Devices

In order to investigate the steric effects of the three new Ru complexes on their PV characteristics, various concentrations of CDCA (in the range of 0–20 mM) were added into the dye solutions for the devices. As displayed in Figure S4, the relative deviation in the PV parameters [*J*_sc_, *V*_oc_, fill factor (FF), and PCE] is measured under the STC for the cells based on **CYC-B23C** and **CYC-B23T**, and the *J*_sc_ values of both cells linearly decline with the increased addition of CDCA. Meanwhile, their deviation in *V*_oc_ and FF (within ±1.5%) cannot compensate for the *J*_sc_ loss. In consequence of the addition of CDCA, the PCE of the devices sensitized with the two stereoisomers decreases. More importantly, the highest PCE occurred in the absence of CDCA, indicating that the two stereoisomers with a hexyl-thienylene-vinylene moiety in the anchoring ligand display a steric effect on reducing the dye aggregation, thereby having a potential as the coadsorbent-free dyes for DSC. On the other hand, the deviation for the **CYC-B22**-sensitized devices with various concentrations of CDCA is less than ±2.8%, nearly independent of the presence of CDCA. These results also confirm that **CYC-B22** showing moderate steric effects is a promising candidate for the coadsorbent-free DSC, attributed to the anchoring ligand functionalized with dual hexyl-thienylene-vinylene moieties, and the two anchoring sites triumph in the competition of adsorption (between **CYC-B22** and CDCA).

**Figure 5:**
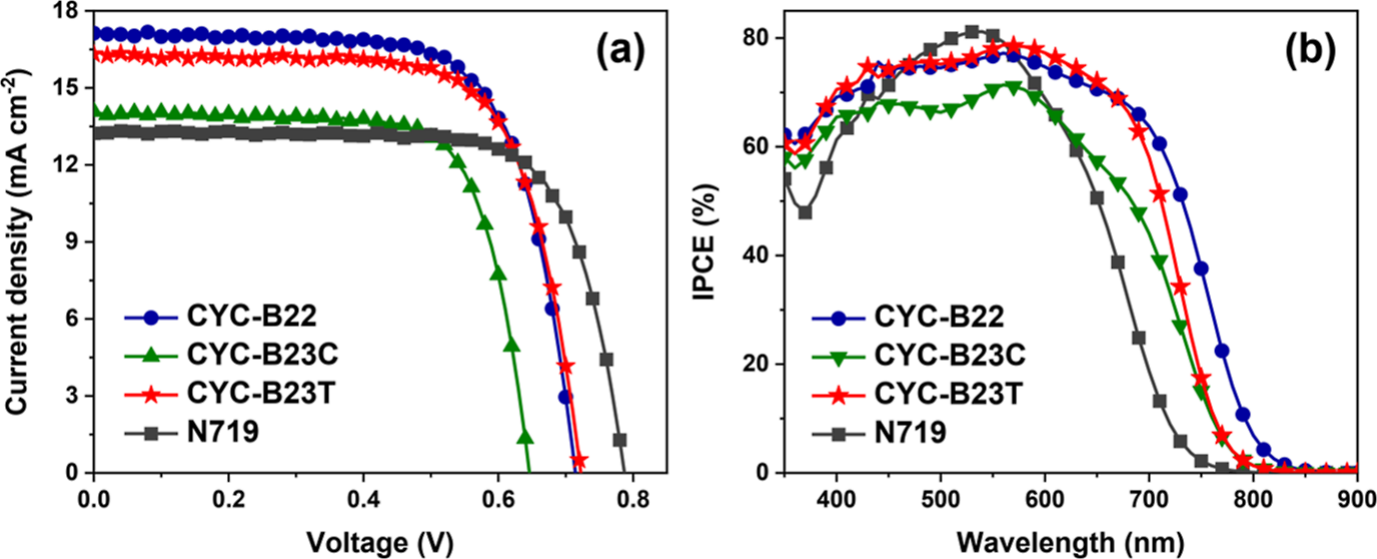
(a) *J*–*V* characteristic curves of the coadsorbent-free devices with the sensitization of various Ru complexes measured under the STC, and (b) the corresponding IPCE spectra.

The *J*–*V* curves of the highest-efficiency coadsorbent-free devices sensitized with **CYC-B22**, **CYC-B23C**, **CYC-B23T**, and **N719** are shown in Figure 5a. The corresponding PV parameters, mean, and standard deviation obtained from total 40 devices dyed with the four Ru complexes are shown in Table 2. The histograms of the PV parameters are presented in Figure S5. For accuracy, the spectral mismatch factors (SMMs)^53^ were calculated for correcting all of the PV parameters. The relevant spectra and the corresponding SMMs are summarized in Figure S6 and Table S2, respectively. Furthermore, the calculated *J*_sc_ values of the devices derived from the IPCE spectra (Figure 5b and Table 2) and the AM 1.5 global standard spectrum^53^ are greatly consistent with those extracted from the *J*–*V* curves (the deviation is lower than 1%). The coadsorbent-free device with the sensitization of **CYC-B22** displays *J*_sc_, *V*_oc_, and FF of 17.13 mA cm^–2^, 0.714 V, and 70.57%, respectively, providing the best PCE of 8.63%. It is noteworthy that the *J*_sc_ and PCE are superior to most of the other heteroleptic Ru sensitizers bearing conjugation-extended bpy anchoring ligands for DSC (Table S1). In contrast, the cells sensitized with **CYC-B23C** and **CYC-B23T** display *J*_sc_, *V*_oc_, FF, and PCE of 14.08 mA cm^–2^, 0.646 V, 73.05%, and 6.64% and 16.32 mA cm^–2^, 0.722 V, 71.11%, and 8.38%, respectively. The distinct difference in the *J*_sc_ values of the devices can be explained by the IPCE spectra (Figure 5b). It is shown that the device sensitized with **CYC-B22** displays a panchromatic response extending to 850 nm, which is broader than those of the cells based on **CYC-B23C**, **CYC-B23T**, **N719**, and the other bipyridyl heteroleptic Ru complexes using dcbpy anchoring ligand.^1,2,15,16,30−36,47^ Evidently, the highest *J*_sc_ and PCE of **CYC-B22**-sensitized device are ascribed to the best light-harvesting capability provided by the highly conjugated anchoring and ancillary ligands as well as the highest amount of dye-loading (77.8 nmol cm^–2^ in Table 2) for the largest light-harvesting efficiency^17^ (LHE; Figure S7). Surprisingly, the PCE of the **CYC-B23T**-sensitized device is comparable with that of the **CYC-B22**-based cell but remarkably higher than those of cells dyed with its stereoisomer (**CYC-B23C**). It is noted that the IPCE response in the wavelength of 350–700 nm for the **CYC-B23T**-sensitized device is also higher than that of **CYC-B23C**. Furthermore, the dye-loadings of **CYC-B23C** and **CYC-B23T** in the mesoscopic TiO_2_ anode are 72.2 nmol cm^–2^ and 63.5 nmol cm^–2^, respectively (Table 2), confirming that the unsymmetrical anchoring ligand induces different adsorptions for the two stereoisomers on TiO_2_. The LHE of **CYC-B23C** is marginally superior to that of **CYC-B23T** (Figure S7), which is inconsistent with their IPCE spectra. It is noted that their absorption on TiO_2_ (see Figure S1) also differs from the IPCE spectra. These results hint that the remarkable discrepancy in the PV parameters and spectral responses between the two stereoisomers should be mainly due to the steric effects created by the conjugated and unsymmetrical anchoring ligand on the intermolecular interactions of the Ru complexes adsorbed on TiO_2_, beyond their similarity in electronic transitions and driving forces for electron injection and dye regeneration.

**Table 2:** PV Parameters and Dye-Loading of the Coadsorbent-Free Devices with the Sensitization of Various Ru Complexes^a^

Ru complex	calc. *J*_sc_ (mA cm^–2^)^b^	*J*_sc_ (mA cm^–2^)	*V*_oc_ (V)	FF (%)	PCE (%)	dye-loading (nmol cm^–2^)
**CYC-B22**	17.17	17.13	0.714	70.57	8.63	77.8
		(16.08 ± 0.58)	(0.719 ± 0.004)	(71.83 ± 1.16)	(8.31 ± 0.23)	
**CYC-B23C**	14.13	14.08	0.646	73.05	6.64	72.2
		(13.42 ± 0.56)	(0.648 ± 0.006)	(72.96 ± 0.69)	(6.34 ± 0.24)	
**CYC-B23T**	16.38	16.32	0.722	71.11	8.38	63.5
		(15.83 ± 0.42)	(0.726 ± 0.008)	(71.26 ± 0.75)	(8.19 ± 0.19)	
**N719**	13.31	13.24	0.787	74.37	7.75	—
		(13.50 ± 0.38)	(0.769 ± 0.013)	(72.69 ± 0.91)	(7.55 ± 0.16)	

a The values within parentheses represent the mean and standard deviation calculated from 10 devices.

b The values are computed based on the IPCE spectra of the devices and the AM 1.5 global standard spectrum.

### 3.4 Electron Injection Efficiency, Dye Regeneration Kinetics, and CR Dynamics

To explore the effects induced by the highly conjugated anchoring ligands and stereoisomerism on the distinct PV parameters of the cells with the sensitization of **CYC-B22**, **CYC-B23C**, and **CYC-B23T** (especially the latter two stereoisomers), the electron injection efficiency, dye regeneration kinetics, and CR dynamics for the devices were scrutinized. For injection efficiency, the time-correlated single photon counting technique (TCSPC)^57,58^ was used to obtain the excited state lifetimes of the Ru complexes in degassed DMF solution. Moreover, the emission lifetime image method^59^ was employed to measure the lifetimes of the Ru complexes adsorbed on 12 μm thick transparent TiO_2_ films. As summarized in Table S3, the electron injection efficiency of **CYC-B22** (89.5%), **CYC-B23C** (91.7%), and **CYC-B23T** (87.9%) is similar and comparable with that of **N719** (90.9%). These results confirm that incorporating carboxylated hexyl-thienylene-vinylene units into the anchoring ligands does not adversely affect the electron injection process, despite the increased distance between the Ru-NCS mixed orbitals and the surface of TiO_2_ due to the presence of conjugated moieties.

**Figure 6:**
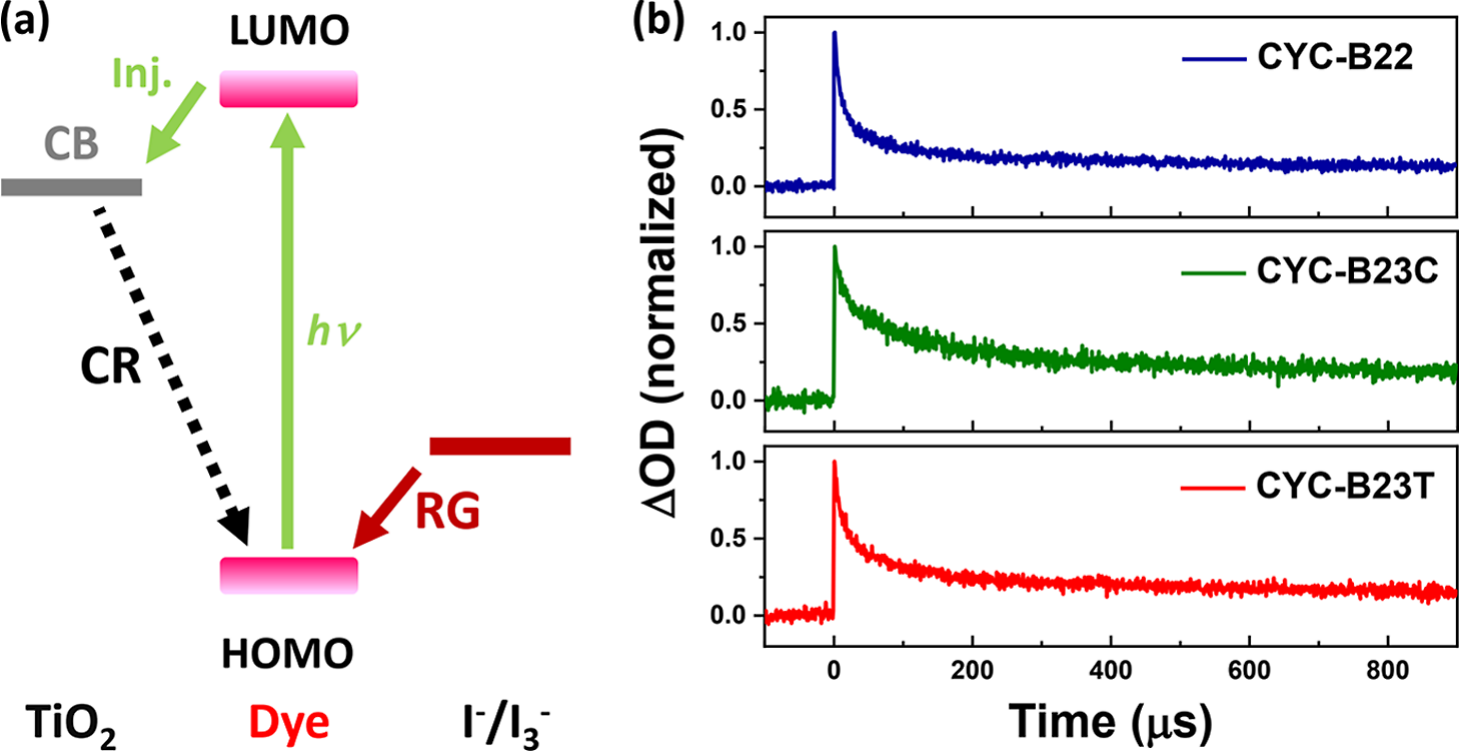
(a) Schematic presentation of two pathways (RG and CR) for reducing the photo-oxidized Ru complexes in devices. (b) Transient absorption spectra of the coadsorbent-free cells with the sensitization of the new Ru complexes and the iodide electrolyte.

As illustrated in Figure 6a, both of the electrons transferred from iodide electrolyte and TiO_2_ can reduce the photo-oxidized Ru complexes. The former is the favored dye regeneration (RG), and the latter case is undesired CR. The kinetics of the two electron transferring pathways in the devices sensitized with the three new Ru complexes were measured with transient absorption spectroscopy (TAS).^17^ The spectra and the decay half-times (*t*_50%_-RG associated with the reduction of the photo-oxidized Ru complexes through iodide electrolyte, and *t*_50%_-CR for the electrons transferred from TiO_2_ anode) are presented in Figure 6b and Table 3, respectively. The order of the *t*_50%_-RG for the new Ru complexes is **CYC-B22** (8.3 μs) < **CYC-B23C** (11.6 μs) < **CYC-B23T** (11.8 μs), suggesting that the flexibility of hexyl groups linked in both of the anchoring and ancillary ligands is beneficial for the charge transfer from iodide to the photo-oxidized Ru complexes. On the other hand, the order of the *t*_50%_-CR for the Ru complexes is **CYC-B22** (115.5 μs) < **CYC-B23C** (132.7 μs) < **CYC-B23T** (133.5 μs). These results indicate that the electron donation from iodide is dominant in the regeneration of the oxidized Ru complexes. Furthermore, the noteworthy ratios between the half-times (*t*_50%_-RG/*t*_50%_-CR) of **CYC-B22**, **CYC-B23C**, and **CYC-B23T** are 75/25, 49/51, and 67/33, respectively, implying that the devices based on **CYC-B22** and **CYC-B23T** have the probabilities of CR lower than that of **CYC-B23C**, which contributes to the higher PCE of the devices.

**Table 3:** Electron Transferring Half-Times and the Relative Ratio for RG and CR Pathways of Reducing Photo-Oxidized Ru Complexes in Coadsorbent-Free Devices

complex	*t*_50%_-RG (μs)	*t*_50%_-CR (μs)	ratio of *t*_50%_-RG and *t*_50%_-CR (*R*_RG_/*R*_CR_)
**CYC-B22**	8.3	115.5	75/25
**CYC-B23C**	11.6	132.7	49/51
**CYC-B23T**	11.8	133.5	67/33

**Figure 7:**
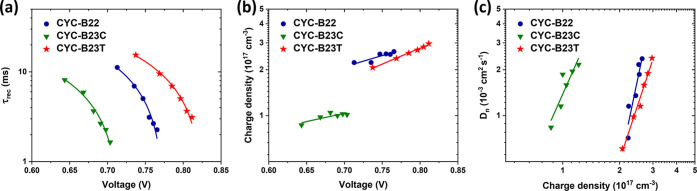
(a) CR lifetime (τ_rec_) versus voltage, (b) charge density versus voltage, and (c) diffusion coefficient (*D*_n_) versus charge density for the coadsorbent-free devices sensitized with **CYC-B22**, **CYC-B23C**, and **CYC-B23T**, respectively.

The photoelectrical transient measurements, including IMVS/IMPS and charge extraction^17,54−56^ were performed for more insights into the charge transfer dynamics in the devices. As depicted in Figure 7a, the CR lifetime (τ_rec_) of the devices extracted from IMVS escalates in a sequential order (**CYC-B23C** < **CYC-B22** < **CYC-B23T**), which well matches with the sequence of the cells’ *V*_oc_ (Table 2). The charge density at the TiO_2_ electrodes as a function of voltage obtained from charge extraction measurements (Figure 7b) shows that the devices based on **CYC-B22** and **CYC-B23T** have more charge accumulation in the TiO_2_ than that dyed with **CYC-B23C**, which is contradictory to the tendency of the *V*_oc_. The charge density dependence of the diffusion coefficient (*D*_n_) estimated from IMPS (Figure 7c) shows the same trend as that in Figure 7b. The less charge accumulation of **CYC-B23C** dyed TiO_2_ than those of the other two dyes suggests less surface trap sites in the TiO_2_ and therefore the higher *D*_n_ than those of **CYC-B22** and **CYC-B23T**. The consolidated results from Figure 7b,c demonstrate that the conduction band (or quasi Fermi level) of **CYC-B23C**-adsorbed TiO_2_ is higher than those of the other two dyes so that the intrinsic *V*_oc_ should be larger in the **CYC-B23C**-based cell. However, the data in Figure 7a show that the ability of suppressing CR following the order of **CYC-B23C** < **CYC-B22** < **CYC-B23T**, which manifests that the dominant factor to determine the *V*_oc_ of the devices is the capability of inhibiting the CR induced by the steric effects of the anchoring ligands. For a deeper understanding of the *J*–*V* characteristics, series resistance (*R*_s_), diode ideality factor (*n*), and shunt resistance (*R*_sh_)^4^ were calculated for the best devices sensitized with three new Ru complexes (Figure 5a). It is noted that the *R*_s_ is in the order of **CYC-B23C** (5.0 Ω cm^2^) < **CYC-B22** (5.2 Ω cm^2^) < **CYC-B23T** (5.4 Ω cm^2^); meanwhile, the *n* and *R*_sh_ based on **CYC-B23C**, **CYC-B22**, and **CYC-B23T** are 1.023 and 4058 Ω cm^2^, 1.265 and 4205 Ω cm^2^, and 1.258 and 5981 Ω cm^2^, respectively. The *R*_s_ and *n* values explain the order of FF for the devices (**CYC-B22** < **CYC-B23T** < **CYC-B23C**), while the order of *R*_sh_ (**CYC-B23C** < **CYC-B22** < **CYC-B23T**) is consistent with the τ_rec_ and *V*_oc_, which also accentuates the capability of **CYC-B23T** to suppress the CR in the cell.

### 3.5 DFT Simulation of Ru Complexes Adsorbed on TiO_2_

**Figure 8:**
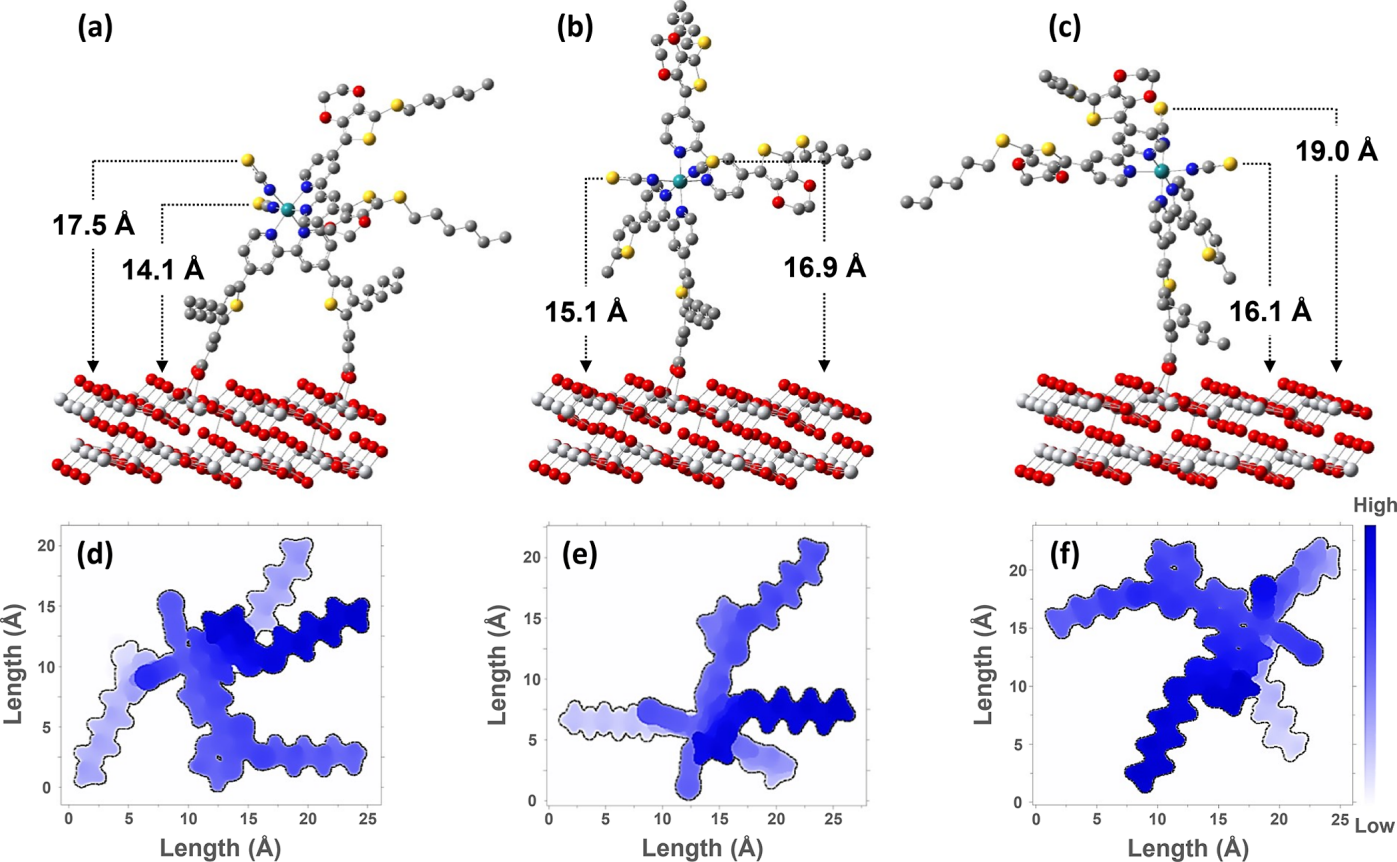
Molecular geometries (protons are omitted for clarity) and the projection areas of (a,d) **CYC-B22**, (b,e) **CYC-B23C**, and (c,f) **CYC-B23T** adsorbed on the surface of the anatase (TiO_2_)_64_ unit cell. The color scale indicates the relative vertical distance of the atoms in the complexes from the TiO_2_ surface. Darker colors represent atoms that are positioned at a considerable distance from the surface of TiO_2_, while lighter colors indicate atoms that are closer in proximity.

It was reported that the bpy-based anchoring ligands of Ru complexes affect the molecular adsorption geometry and the interfacial electron transfer;^60^ meanwhile, the NCS ligands play a crucial role in facilitating surface-confined hole transportation.^61^ Moreover, the sulfur atoms of the NCS ligands can interact with iodine in the electrolyte, thereby increasing CR and reduction of triiodide around the TiO_2_ surface.^1,62,63^ For the distinct difference of the steric effects in the PV characteristics and CR lifetime (τ_rec_) of the devices based on the three new Ru complexes all having two NCS ligands (which are highly involved in the HOMOs as the EDDMs provided in Figure 4b–d), it is speculated that the geometric structures of the complexes adsorbed on the surface of TiO_2_ should be critical. Therefore, their molecular geometry on the anatase (TiO_2_)_64_ unit cell through the bridged bidentate binding mode^64^ and the (101) surface of the TiO_2_^65^ was optimized by DFT as shown in Figure 8. In the case of **CYC-B22** (Figure 8a), the distances between the two sulfur atoms of the NCS ligands and the surface of the (TiO_2_)_64_ unit cell are 14.1 and 17.5 Å, respectively. In the two stereoisomers, the distances for **CYC-B23C** (Figure 8b) are 15.1 and 16.9 Å, while those for **CYC-B23T** (Figure 8c) are 16.1 and 19.0 Å. In addition, Figure 8d–f depicts the projection images of the three complexes adsorbed on the (TiO_2_)_64_ unit cell, obtained using Multiwfn software.^66^ The estimated areas, determined using ImageJ software,^67^ were found to be 179.0, 140.7, and 170.0 Å^2^, respectively. These values correspond to relative surface passivation, calculated with the dye-loadings of 100, 73.0, and 77.6% for **CYC-B22**, **CYC-B23C**, and **CYC-B23T**, respectively. These findings indicate that the CR, resulting from the shortest distance of **CYC-B22** could be diminished by the largest surface passivation. More importantly, the longest distance in the system of **CYC-B23T** not only offsets the lower surface passivation but also leads the devices to the longest τ_rec_ and thus the highest *V*_oc_ among the three Ru complexes dyed DSCs. In other words, the molecular simulation clarifies that the steric effects induced by different geometric structures of the adsorption for **CYC-B23T** and **CYC-B23C** (having the same unsymmetrical anchoring ligand) on the TiO_2_ are the dominant factors for the distinguishable PV performance of the cells. Moreover, these findings further signify that the highly conjugated and unsymmetrical anchoring ligand shows promise, not only in the enhancement of the absorption capability for the Ru complexes but also in the elongation of the distance between the NCS ligands and the TiO_2_ surface for inhibiting the undesired CR in the devices.

### 3.6 Ru Complexes–Iodine Binding Constants and Device Stability

**Figure 9:**
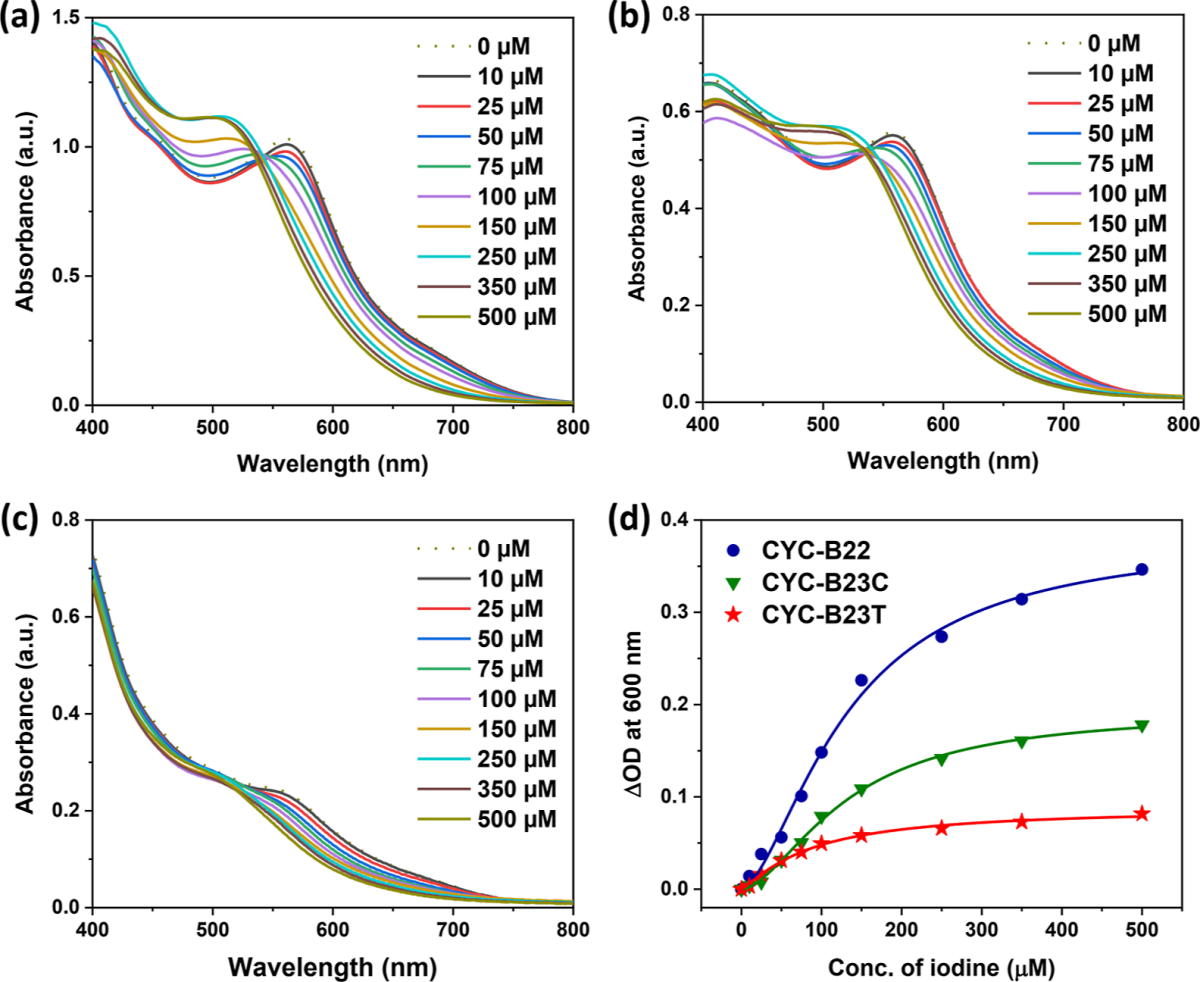
Absorption spectra of (a) **CYC-B22**, (b) **CYC-B23C**, and (c) **CYC-B23T** adsorbed on 4 μm thick TiO_2_ films and immersed in acetonitrile with various concentrations of iodine. Alterations in the absorption spectra of the solutions have been deducted. (d) Variations in absorbance at 600 nm (ΔOD = −(*A* – *A*_0_)) plotted against the concentration of iodine for TiO_2_ films based on various Ru complexes.

It was reported that iodine binding to dyes could affect not only the electronic transitions of the dyes but also the performance of the devices.^62,63,68,69^ To examine the difference in iodine bindings between the three new Ru complexes, their adsorbed transparent TiO_2_ thin films were immersed in acetonitrile containing various concentrations of iodine (in the range of 0–500 μM) for the measurement of absorption spectra. As displayed in Figure 9a, the λ_max_ of the **CYC-B22**-adsorbed TiO_2_ film hypsochromically shifts (from 561 nm to ca. 505 nm) when the concentration of iodine successively increases. Similar scenarios are observed in the **CYC-B23C**-adsorbed film (Figure 9b; λ_max_ shifts from 558 nm to ca. 500 nm) and the previous study on Ru complexes.^63^ Nevertheless, the variation of absorption profiles of the **CYC-B23T**-sensitized film (Figure 9c) is significantly distinct from those of the former two. For quantitative analysis, the changes in OD at 600 nm vs iodine concentration and the fitting curves based on Langmuir isotherm equation^63^ for iodine binding constants are summarized in Figure 9d. **CYC-B22** and **CYC-B23C** adsorbed on TiO_2_ films show the binding of 3.6 (±1.7) × 10^3^ M^–1^ and 2.4 (±1.1) × 10^3^ M^–1^, whereas that for **CYC-B23T** is 46.4 (±19) × 10^3^ M^–1^. The highest iodine binding constant of **CYC-B23T** induces bleaching of the adsorbed TiO_2_ film, which should be due to the large free space around NCS ligands (as seen in Figure 8c,f). However, it is surprising that the iodine binding of **CYC-B23T** does not result in any detrimental impact on the performance of the devices, which might be ascribed to the magnitude limited by the lower dye loading (Table 2) and the longest distance between one of the NCS ligands and the TiO_2_ surface (Figure 8c) for suppressing the CR in the devices.

**Figure 10:**
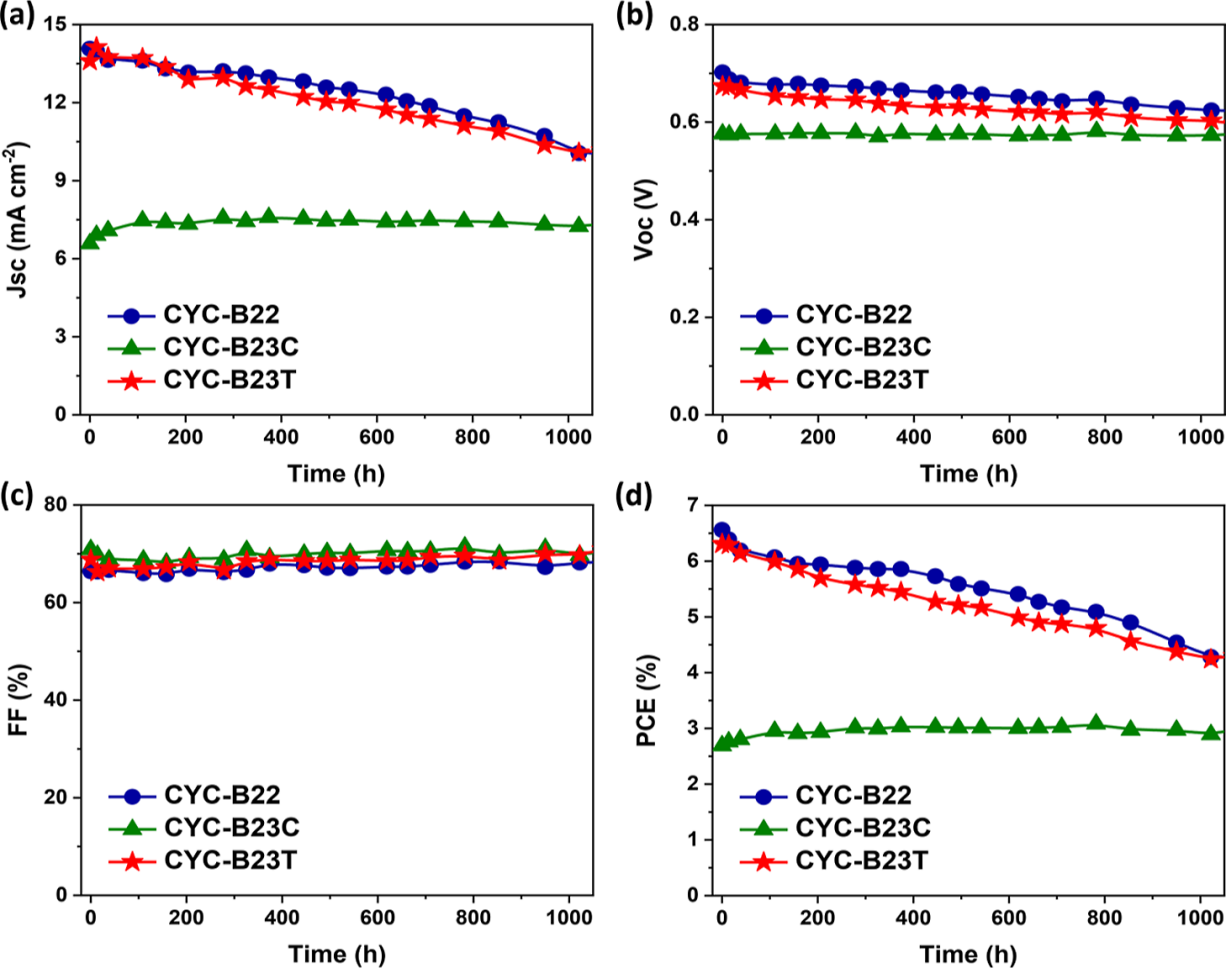
Evolution of (a) *J*_sc_, (b) *V*_oc_, (c) FF, and (d) PCE for devices sensitized with **CYC-B22**, **CYC-B23C**, and **CYC-B23T**, respectively. The devices were stored at a temperature of 26 ± 4 °C and a relative humidity of 75 ± 10% and measured under the STC.

To fully explore the steric effects resulting from the highly conjugated and unsymmetrical anchoring ligands on the PV performance of the Ru complexes, the stability of the devices using a low-volatility electrolyte was tested. Figure 10 depicts the change in the PV parameters. The devices sensitized with **CYC-B22** and **CYC-B23T** initially show a comparable PCE (6.56% vs 6.31%), which is remarkably higher than that of **CYC-B23C** (2.69%). After storage (temperature of 26 ± 4 °C and relative humidity of 75 ± 10%) for over 1000 h, the *J*_sc_ and *V*_oc_ losses in the devices based on **CYC-B22** and **CYC-B23T** are more pronounced than that of **CYC-B23C**, while the FF of all three devices remains stable. As a consequence, the devices based on **CYC-B22** and **CYC-B23T** still hold the PCE (4.28 and 4.24%, respectively) higher than that of **CYC-B23C** (2.89%); however, it is a fact that the stability of the **CYC-B23C**-sensitized cell is the best among them. To further verify the difference in stability, the devices stored at a temperature of 70 ± 3 °C for around 50 h were also examined (Figure S8). Under thermally accelerated aging, the **CYC-B23C**-sensitized cell also exhibits the best robustness, which should be attributed to the best diode ideality factor for a better alignment of the Ru complex on TiO_2_ and the lowest iodine binding constant for the sustainability of light-harvesting. These results demonstrate that the steric effects on the adsorption orientation of Ru complexes lead the devices to display not only a distinguishable PCE but also a different stability, which can be controlled by the anchoring ligands, in particular the unsymmetrical one.

## 4 Conclusions

Three new heteroleptic Ru complexes (**CYC-B22**, **CYC-B23C**, and **CYC-B23T**) with highly conjugated anchoring and ancillary ligands both featuring thienylenes are prepared to study the impact of electronic and steric factors on the PV performance of the Ru complexes applied in DSCs. Compared with all of the other bpy-based heteroleptic Ru sensitizers bearing conjugation-extended bpy anchoring ligands, **CYC-B22** shows the best light-harvesting capability. The corresponding coadsorbent-free device has a panchromatic response extending to 850 nm for a good PCE of 8.63%. Additionally, the two stereoisomers **CYC-B23C** and **CYC-B23T**, coordinated with an unsymmetrical anchoring ligand, display similar electronic transitions and energy level of the frontier molecular orbitals. Nevertheless, the PCE (6.64% vs 8.38%) and stability of the devices are notably distinguishable. Compared with the cells based on **CYC-B22** and **CYC-B23C**, the longest CR lifetime and the highest *V*_oc_ of the **CYC-B23T**-sensitized device are credited to the longest distance (19.0 Å) between one sulfur atom of the NCS ligands and the surface of the TiO_2_. This study demonstrates that the steric effects of Ru complexes on suppressing dye aggregation and CR can be well-controlled by the functionalized anchoring ligand and stereoisomerism for realizing coadsorbent-free, panchromatic, and efficient DSC. Moreover, by finely tuning the structure of unsymmetrical anchoring ligands, we can deliberately adjust the adsorption geometry and iodine bonding of metal complexes on the TiO_2_ surface, which consequently impacts the performance and stability of the devices. This work paves a new way for the innovation of polypyridyl metal complex sensitizers for DSC applications.

## Supporting Information Available

Synthesis of esterified anchoring ligands, summarized absorption properties and device performance of other Ru complexes bearing conjugation-extended bpy anchoring ligands, square-wave voltammograms, CDCA effects on PV parameters of devices, histograms of PV parameters, spectra for spectral mismatch factors, light-harvesting efficiency spectra, electron injection efficiency, and stability of devices under thermally accelerated aging for the Ru complexes (PDF)
