# Supplementary material for: Steric Effects on the Photovoltaic Performance of Panchromatic Ruthenium Sensitizers for Dye-Sensitized Solar Cells
Source: ACS Appl Mater Interfaces. 2024 Mar 4;16(10):12647–60. doi: 10.1021/acsami.3c19298 (PMC10941073; doi:10.1021/acsami.3c19298)
Supplement: Supplementary file 1 — am3c19298_si_001.pdf [file am3c19298_si_001.pdf]

Supporting Information

Steric Effects on Photovoltaic Performance of Panchromatic Ruthenium Sensitizers for Dye-Sensitized Solar Cells

Chia-Yuan Chen,[*,†,‡] Ting-Yi Lin,[‡] Chi-Feng Chiu,[‡] Mandy M. Lee,[§] Wei-Long Li,[‡] Min-Yu Chen,[‡] Tzu-Hao Hung,[‡] Zhao-Jie Zhang,[‡] Hui-Hsu Gavin Tsai,[*,†,‡] Shih-Sheng Sun,[*,§] and Chun-Guey Wu[*,†,‡]

[†]Research Center of New Generation Light Driven Photovoltaic Modules and [‡]Department of Chemistry, National Central University, Taoyuan 32001, Taiwan, R.O.C.

E-mail address of Professor Chia-Yuan Chen: chiayuan@ncu.edu.tw

E-mail address of Professor Hui-Hsu Gavin Tsai: hhtsai@cc.ncu.edu.tw

E-mail address of Professor Chun-Guey Wu: t610002@cc.ncu.edu.tw

[§]Institute of Chemistry, Academia Sinica, No. 128, Academia Road, Sec. 2, Nankang, Taipei, 115, Taiwan, R.O.C.

E-mail address of Professor Shih-Sheng Sun: sssun@chem.sinica.edu.tw

1. Synthetic Procedures of Esterified Anchoring Ligands (L22-ester and L23-ester)

Synthesis of 2-bromo-3-hexylthiophene (2)

4.72 g (28.05 mmol) 3-hexylthiophene (**1**) prepared according to the literature[S1] was mixed with 4.00 g (22.47 mmol) *N*-bromosuccinimide (NBS) in 80 mL anhydrous DMF under Argon at 0 °C. After stirring the mixture under Argon at room temperature (RT) for 4 h, 30 mL $HCl_{(aq.)}$ (1.2 M) was added to quench the reaction. The mixture was diluted with dichloromethane (DCM) and then successively extracted with saturated $NaHCO_{3(aq.)}$, deionized water, and saturated $NaCl_{(aq.)}$. The organic phase was collected, dried over $MgSO_4$, filtered and concentrated under reduced pressure. The crude product was purified using silica gel chromatography with hexane (Hxn) as an eluent to afford 2-bromo-3-hexylthiophene (**2**) as a colorless liquid (4.72 g, 85.0% yield). ^{1}H-NMR (300 MHz, δ/ppm in $CDCl_3$): 7.23 (d, *J* = 4.8 Hz, 1H), 6.91 (d, *J* = 4.8 Hz, 1H), 2.61 (t, *J* = 7.5Hz, 2H), 1.62 (m, 2H), 1.35 (m, 2H), 1.30 (m, 4H), 0.90 (t, *J* = 7.2 Hz, 3H).

Synthesis of 3-hexylthiophene-2-carbaldehyde (3)

5.20 g (21.04 mol) 2-bromo-3-hexylthiophene (**2**) and 0.62 g (25.5 mmol) Mg powder in 40 mL anhydrous tetrahydrofuran (THF) were mixed with 0.84 g (3.31 mmol) I_2 in 20 mL anhydrous THF. After refluxing at 70 °C for 5 h then cooling to RT, 8 mL anhydrous DMF was added into the mixture and the solution was stirred under Argon at RT for 8 h. 30 mL $HCl_{(aq.)}$ (1.2 M) was added to quench the reaction, ether was used to dilute the mixture. The organic phase was successively extracted with saturated $Na_2CO_{3(aq.)}$ and deionized water. then dried over $MgSO_4$, filtered and concentrated under reduced pressure. The crude product was purified using silica gel chromatography with ethyl acetate (EA)/Hxn = 1/20 as an eluent to afford 3-hexylthiophene-2-carbaldehyde (**3**) as a yellowish liquid (2.61 g, 63.2% yield). ^{1}H-NMR (300 MHz, δ/ppm in $CDCl_3$): 10.02 (s, 1H), 7.62 (d, *J* = 4.8 Hz, 1H), 6.99 (d, *J* = 4.8 Hz, 1H), 2.95 (t, *J* = 7.5 Hz, 2H), 1.65 (m, 2H), 1.35 (m, 2H), 1.27 (m, 4H), 0.87 (t, *J* = 7.2 Hz, 3H).

Synthesis of 2-(3-hexylthiophen-2-yl)-1,3-dioxolane (4)

1.80 g (9.17 mmol) 3-hexylthiophene-2-carbaldehyde (**3**), 0.52 g (2.73 mmol) *p*-toluenesulfonic acid monohydrate (TsOH·H_2O), 17.0 g (273 mmol) ethlyene glycol (EG) and 100 mL benzene were mixed and refluxed using a Dean-Stark apparatus at 110 °C for 24 h. After cooling the mixture to RT, saturated $NaHCO_{3(aq.)}$ and DCM were added to terminate the reaction and dilute the mixture, respectively. The organic layer was collected, dried over $MgSO_4$, filtered and concentrated under reduced pressure. The crude product, 2-(3-hexylthiophen-2-yl)-1,3-dioxolane (**4**), obtained as a brownish liquid (2.09 g, 95.0% yield) was used without further purification. ^{1}H-NMR (300 MHz, δ/ppm in $CDCl_3$): 7.12 (d, *J* = 4.8 Hz, 1H), 6.77 (d, *J* = 4.8 Hz, 1H), 6.05 (s, 1H), 4.17 (m, 2H), 3.92 (m, 2H), 2.7 (t, *J* = 7.5 Hz, 2H), 1.65 (m, 2H), 1.35 (m, 2H), 1.27 (m, 4H), 0.87 (t, *J* = 7.2 Hz, 3H).

Synthesis of (5-(1,3-dioxolan-2-yl)-4-hexylthiophen-2-yl)trimethylstannane (5)

4.39 mL *n*-butyllithium (*n*-BuLi; 2.5 M in Hxn) was added slowly under Argon into the solution of 2.20 g (9.15 mmol) 2-(3-hexylthiophen-2-yl)-1,3-dioxolane (**4**) in 40 mL anhydrous THF at -78 °C. After stirring the mixture at -78 °C for 2 h, 2.37 g (11.89 mmol) trimethyltin chloride (TMeSnCl) in 15 mL anhydrous THF was added into the solution at -78 °C. The mixture was stirred at RT for 8 h, followed by an extraction using DCM and saturated $NH_4Cl_{(aq.)}$. The organic phase was dried over $MgSO_4$, filtered and concentrated under reduced pressure to afford 3.51 g crude product (5-(1,3-dioxolan-2-yl)-4-hexylthiophen-2-yl)-trimethylstannane (**5**) with *ca.* 95.0% yield identified with ^{1}H-NMR spectrum. This intermediate was used without further purification.

Synthesis of 5-(2-(4-(5-formyl-4-hexylthiophen-2-yl)pyridin-2-yl)pyridin-4-yl)-3-hexylthiophene-2-carbaldehyde (6)

The solution consisting of 3.50 g (8.68 mmol) (5-(1,3-dioxolan-2-yl)-4-hexylthiophen-2-yl)-trimethylstannane (**5**), 1.23 g (3.92 mmol) 4,4'-dibromo-2,2'-bipyridine (dbbpy) and 0.50 g (0.43 mmol) tetrakis(triphenylphosphine)palladium(0) ($Pd(PPh_3)_4$) in 100 mL anhydrous DMF was refluxed at 140 °C for 36 h. After cooling the solution to RT, saturated $NH_4Cl_{(aq.)}$ was added to terminate the

reaction, followed by adding chloroform ($CHCl_3$) to dilute the solution. The organic phase was successively extracted with saturated $NaHCO_{3(aq.)}$, deionized water, and saturated $NaCl_{(aq.)}$. The organic phase was collected, filtered, concentrated under reduced pressure and then diluted with THF. 30 mL $HCl_{(aq.)}$ (1.2 M) was added into the THF solution and then stirred at RT for 8 h to remove the protecting groups. The crude product was filtered and washed with Hxn to afford 5-(2-(4-(5-formyl-4-hexylthiophen-2-yl)pyridin-2-yl)pyridin-4-yl)-3-hexylthiophene-2-carbaldehyde (**6**) as a yellowish solid (1.08 g, 50.5% yield). ^{1}H-NMR (300 MHz, δ/ppm in $CDCl_3$): 10.08 (s, 2H), 8.75 (d, *J* = 5.1 Hz, 2H), 8.73 (dd, *J* = 5.4 Hz, *J* = 1.8 Hz, 2H), 7.57 (dd, *J* = 5.4 Hz, *J'* = 1.8 Hz, 2H), 7.55 (s, 2H), 3.00 (t, *J* = 7.5 Hz, 4H), 1.72 (m, 4H), 1.36 (m, 12H), 0.92 (t, *J* = 7.2 Hz, 6H).

Synthesis of esterified anchoring ligand-22 (L22-ester)

1.05 g (1.93 mmol) 5-(2-(4-(5-formyl-4-hexylthiophen-2-yl)pyridin-2-yl)pyridin-4-yl)-3-hexylthiophene-2-carbaldehyde (**6**) and 1.42 g (4.24 mmol) methoxycarbonylmethylene-triphenylphosphorane ($Ph_3PCHCOOMe$) in 100 mL anhydrous toluene were refluxed at 110 °C for 24 h. After cooling to RT, the solution was filtered and dried under reduced pressure. The crude product was purified using recrystallization with EA to afford ligand-22-ester (**L22-ester**) as a yellowish solid (0.88 g, 69.4% yield). ^{1}H-NMR (300 MHz, δ/ppm in $CDCl_3$): 8.70 (d, *J* = 5.1 Hz, 2H), 8.64 (d, *J* = 1.8 Hz, 2H), 7.86 (d, *J* = 15.6 Hz, 2H), 7.49 (dd, *J* = 5.4 Hz, *J'* = 1.8 Hz, 2H), 7.47 (s, 2H), 6.26 (d, *J* = 15.6 Hz, 2H), 3.81 (s, 6H), 2.73 (t, *J* = 7.5 Hz, 4H), 1.66 (m, 4H), 1.28 (m, 12H), 0.89 (t, *J* = 7.2 Hz, 6H).

Synthesis of 5-(2-(4-bromopyridin-2-yl)pyridin-4-yl)-3-hexylthiophene-2-carbaldehyde (7)

The solution consisting of 2.10 g (5.21 mmol) (5-(1,3-dioxolan-2-yl)-4-hexylthiophen-2-yl)-trimethylstannane (**5**), 1.47 g (4.68 mmol) dbbpy and 0.30 g (0.26 mmol) $Pd(PPh_3)_4$ in 65 mL anhydrous DMF was refluxed at 140 °C for 24 h. After cooling the solution to RT, saturated $NH_4Cl_{(aq.)}$ was added to terminate the reaction, followed by adding chloroform ($CHCl_3$) to dilute the solution. The organic phase was successively extracted with saturated $NaHCO_{3(aq.)}$, deionized water, and saturated $NaCl_{(aq.)}$. The organic phase was collected, filtered, concentrated under reduced pressure, and then diluted with THF. The solution was added into a 20 mL $HCl_{(aq.)}$ (1.2 M) and then

stirred at RT for 8 h to remove the protecting groups. The crude product was filtered and purified using silica gel chromatography with EA/Hxn = 1/5 as an eluent to afford 5-(2-(4-bromopyridin-2-yl)pyridin-4-yl)-3-hexylthiophene-2-carbaldehyde (**7**) as a yellowish solid (1.34 g, 66.7% yield). ^{1}H-NMR (300 MHz, δ/ppm in $CDCl_3$): 10.10 (s, 1H), 8.74 (d, *J* = 5.1 Hz, 1H), 8.69 (s, 2H), 8.54 (d, *J* = 5.1 Hz, 1H), 7.56 (m, 3H), 3.01 (t, *J* = 7.5 Hz, 2H), 1.76 (m, 2H), 1.36 (m, 6H), 0.92 (t, *J* = 7.2 Hz, 3H).

Synthesis of 3-hexyl-5-(2-(4-(5-methylthiophen-2-yl)pyridin-2-yl)pyridin-4-yl)thiophene-2-carbaldehyde (8)

The solution consisting of 1.34 g (3.12 mmol) 5-(2-(4-bromopyridin-2-yl)pyridin-4-yl)-3-hexylthiophene-2-carbaldehyde (**7**), 0.74 g (2.84 mmol) trimethyl(5-methylthiophen-2-yl)stannane prepared according to the literature[S2] and 0.18 g (0.16 mmol) $Pd(PPh_3)_4$ in 80 mL anhydrous DMF was refluxed at 140 °C for 24 h. After cooling the solution to RT, saturated $NH_4Cl_{(aq.)}$ was added to terminate the reaction, followed by adding chloroform ($CHCl_3$) to dilute the solution. The organic phase was successively extracted with saturated $NaHCO_{3(aq.)}$, deionized water, and saturated $NaCl_{(aq.)}$. The organic phase was dried over $MgSO_4$, filtered, concentrated under reduced pressure to afford 3-hexyl-5-(2-(4-(5-methylthiophen-2-yl)pyridin-2-yl)pyridin-4-yl)thiophene-2-carbaldehyde (**8**) as a yellowish solid (1.18 g, 84.6% yield). This intermediate was used without further purification.

Synthesis of esterified anchoring ligand-23 (L23-ester)

1.15 g (2.57 mmol) 3-hexyl-5-(2-(4-(5-methylthiophen-2-yl)pyridin-2-yl)pyridin-4-yl)thiophene-2-carbaldehyde (**8**) and 1.03 g (3.08 mmol) $Ph_3PCHCOOMe$ in 90 mL anhydrous toluene were refluxed at 110 °C for 24 h. After cooling to RT, the solution was filtered and dried under reduced pressure. The crude product was purified using recrystallization with methanol to afford ligand-23-ester (**L23-ester**) as a yellowish solid (0.95 g, 73.5% yield). ^{1}H-NMR (300 MHz, δ/ppm in $CDCl_3$): 8.71 (d, *J* = 5.1 Hz, 1H), 8.67 (d, *J* = 5.1 Hz, 1H), 8.65 (d, *J* = 1.5 Hz, 1H), 8.63 (d, *J* = 1.5 Hz, 1H), 7.88 (d, *J* = 15.6 Hz, 1H), 7.49 (m, 4H), 6.84 (d, *J* = 3.6 Hz, 1H), 6.28 (*J* = 15.6 Hz, 1H),

3.84 (s, 3H), 2.76 (t, J = 7.5 Hz, 2H), 2.58 (s, 3H), 1.75 (m, 2H), 1.33 (m, 6H), 0.90 (t, J = 7.2 Hz, 3H).

2. Supporting Figures and Tables

Table S1. Absorption Properties and the Device Performance of Ru Complexes Featuring Conjugation-Extended Bpy Anchoring Ligands for DSCs

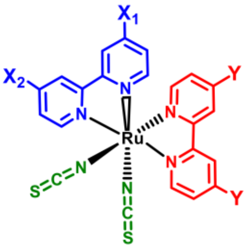

Ru Complex	X_1 and X_2 (wherein Z = H or TBA)	Y	λ_{max} [nm] (ε [10^4 M^{-1} cm^{-1}])	J_{sc} (mA cm^{-2})	V_{oc} (V)	FF (%)	PCE (%)	Co-adsorbent
**K8** (S2)	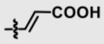		**555 (1.80)** [a]	**18.00**	**0.640**	**75.0**	**8.64**	**w/o**
**BTC-1** (S3)	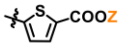		**563 (2.32)** [a]	**15.80**	**0.660**	**73.0**	**7.60**	**w/o**
**K9** (S4)	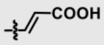	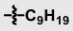	**530 (1.38)** [b]	**15.16**	**0.693**	**65.8**	**6.92**	**w/o**
**K23** (S4)	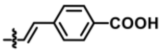	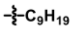	**533 (1.74)** [b]	**15.73**	**0.708**	**66.9**	**7.45**	**w/o**
**BTC-2** (S5)	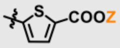	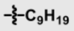	**548 (1.60)** [a]	**16.10**	**0.750**	**74.0**	**9.10**	**GBA**
**1b** (S6)	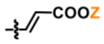	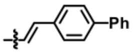	**554 (0.55)** [a]	**5.78**	**0.640**	**68.3**	**2.54**	**w/o**
**1d** (S6)	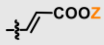	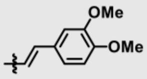	**551 (0.54)** [a]	**4.87**	**0.610**	**65.4**	**1.94**	**w/o**
**H2** (S7)	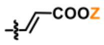	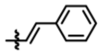	**572 (1.66)** [a]	**4.70**	**0.640**	**68.0**	**2.05**	**w/o**
**CYC-B19** (S9)	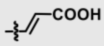	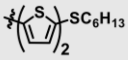	**562 (2.97)** [a]	**16.60**	**0.702**	**72.0**	**8.40**	**DINHOP**
**CYC-B22** [c]	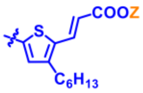	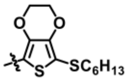	**569 (3.34)** [a]	**17.13**	**0.714**	**70.6**	**8.63**	**w/o**
**CYC-B23C** [c]	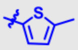 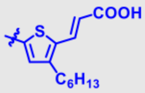	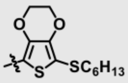	**563 (2.76)** [a]	**14.08**	**0.646**	**73.1**	**6.64**	**w/o**
**CYC-B23T** [c]	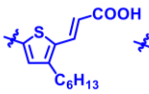 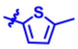	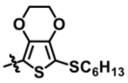	**563 (2.86)** [a]	**16.32**	**0.722**	**71.1**	**8.38**	**w/o**

[a] Measured in DMF; [b] Measured in EtOH; [c] This work.

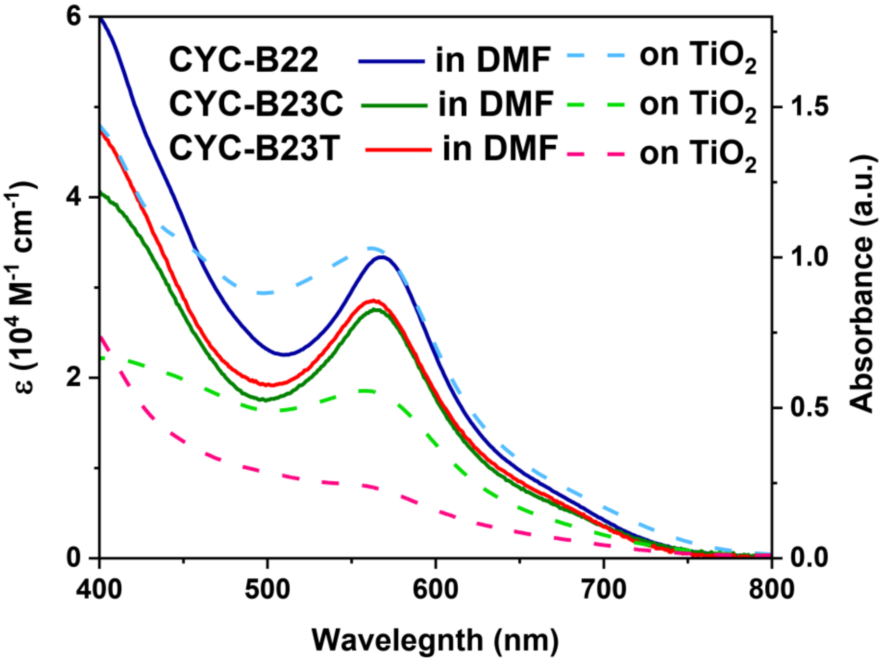

Figure S1. Absorption spectra of **CYC-B22**, **CYC-B23C** and **CYC-B23T** measured in DMF and on TiO_2 thin films (thickness of 4 μm).

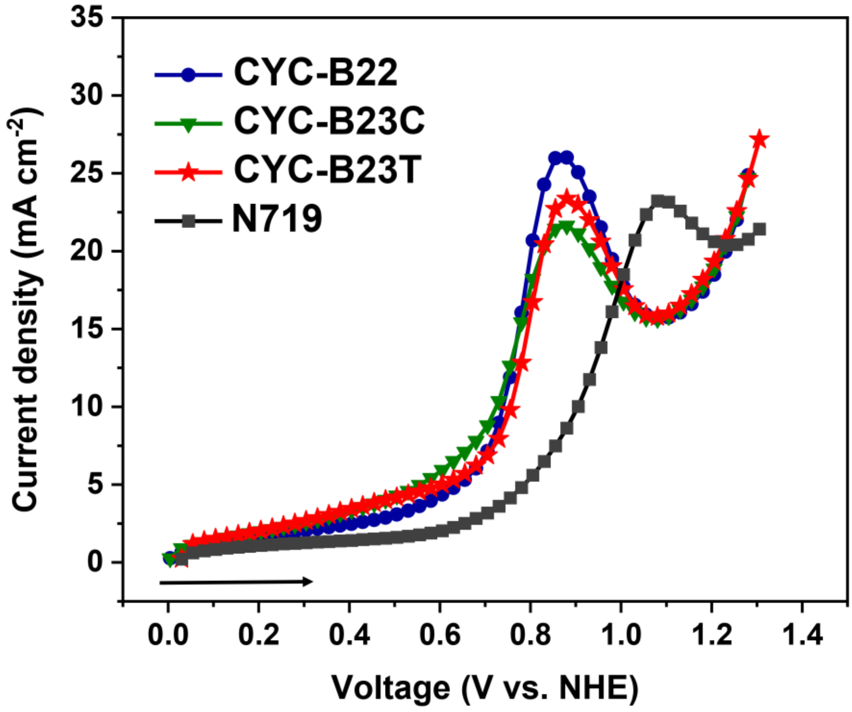

Figure S2. Square-wave voltammograms of the three new Ru complexes and **N719** measured in DMF with 0.1 M tetrabutylammonium perchlorate ($TBAClO_4$) as the supporting electrolyte.

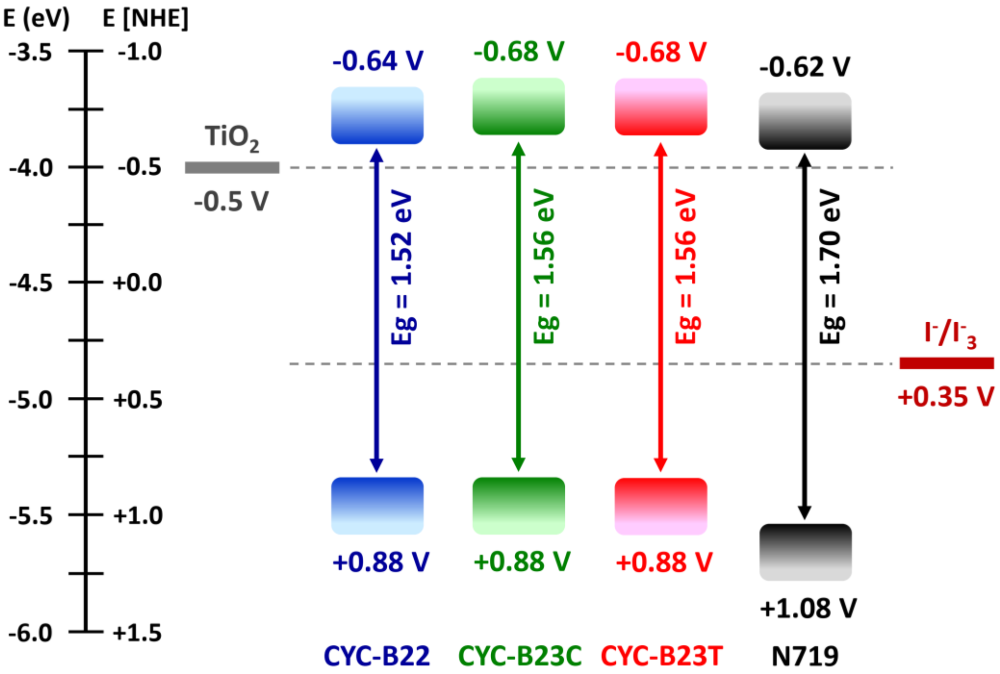

Figure S3. Schematic energy diagram of **CYC-B22**, **CYC-B23C**, **CYC-B23T**, **N719**, TiO_2 and I^-/I_3^-.

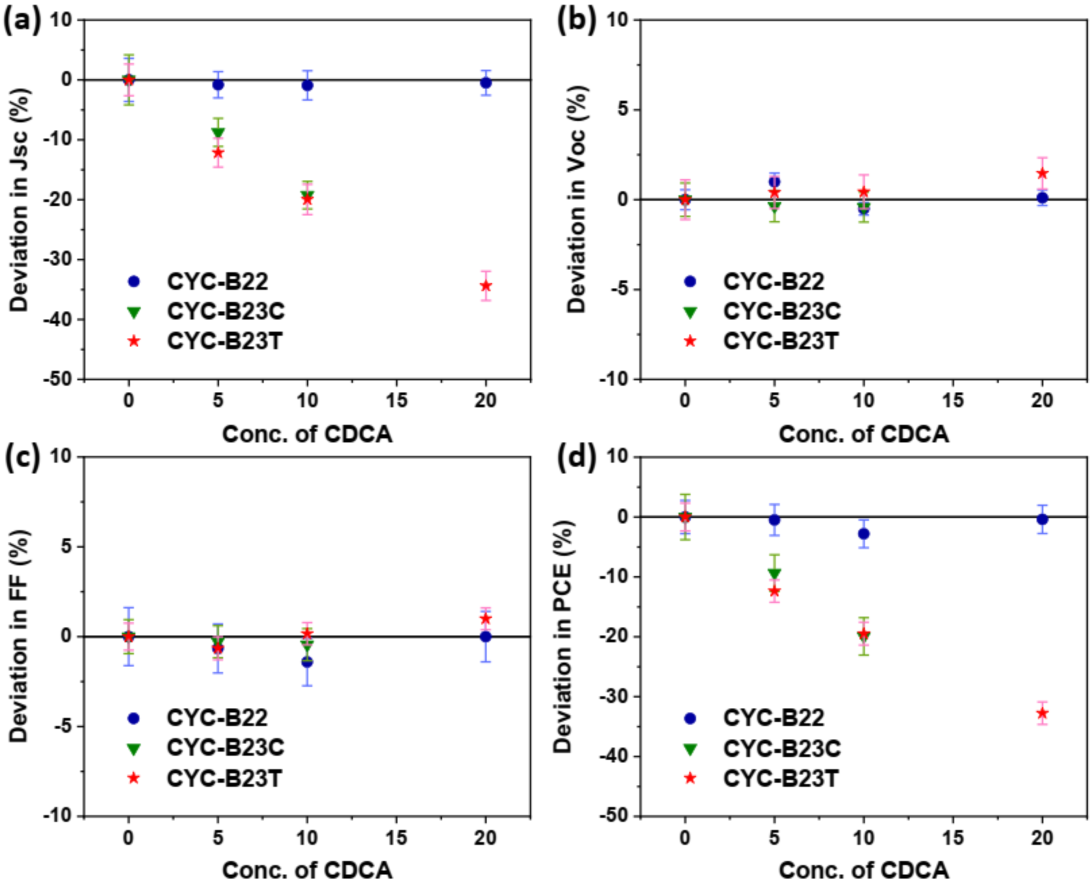

Figure S4. Effects of CDCA on the variation of (a) J_{sc}, (b) V_{oc}, (c) FF, and (d) PCE for the devices sensitized with **CYC-B22**, **CYC-B23C** and **CYC-B23T**. Error bars represent the standard deviation obtained from ten devices without CDCA, and three devices with various concentrations of CDCA.

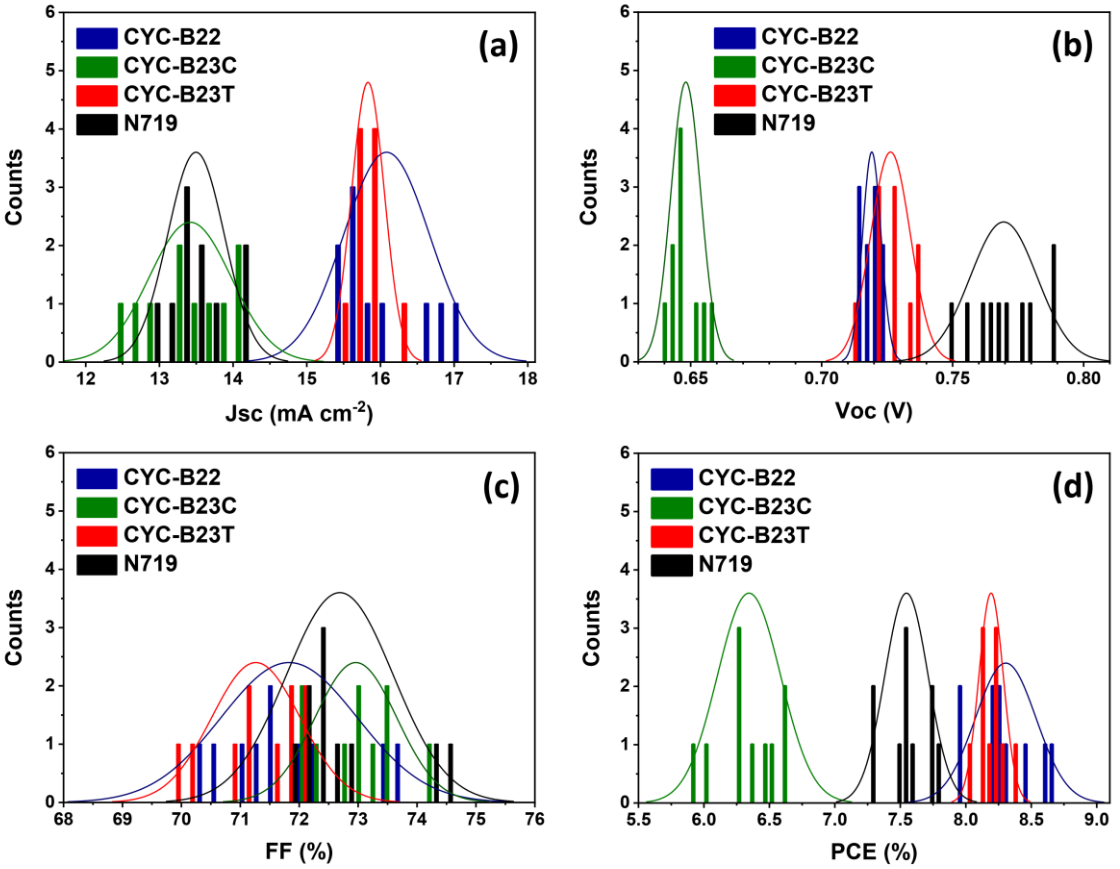

Figure S5. Histograms of (a) J_{sc}, (b) V_{oc}, (c) FF and (d) PCE of the coadsorbent-free devices sensitized with **CYC-B22**, **CYC-B23C**, **CYC-B23T** and **N719**.

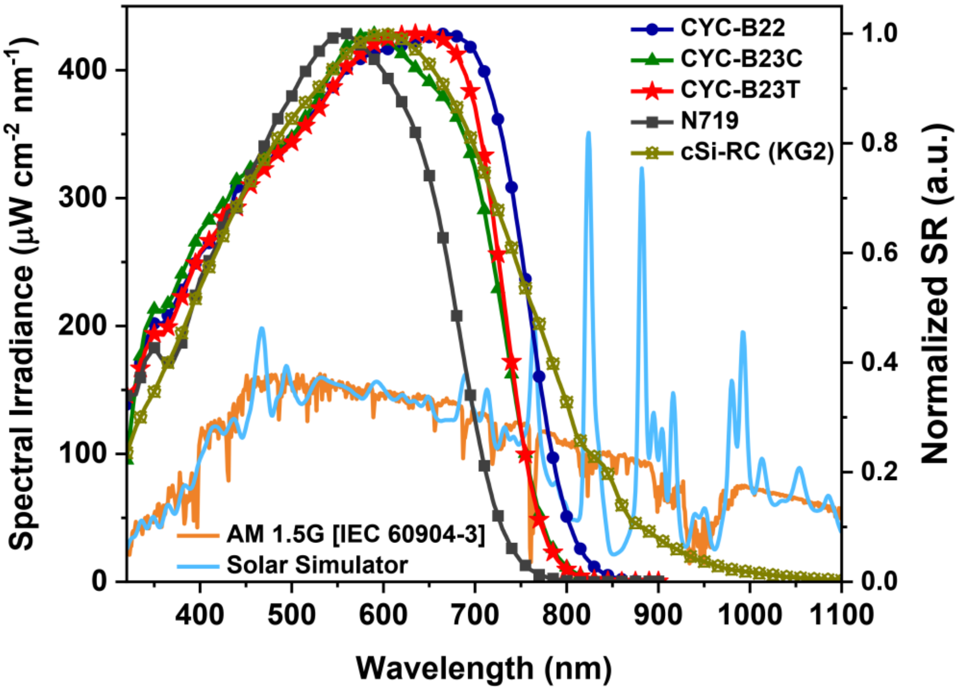

Figure S6. AM 1.5 global standard spectrum, spectral irradiance of the steady-state solar simulator used in this study, as well as the normalized spectral responsivities (SR) of the KG2-filtered single crystalline silicon photovoltaic reference cell (cSi-RC), and the coadsorbent-free devices sensitized with various Ru complexes.

Table S2. Spectral Mismatch Factors (SMMs) of Coadsorbent-Free Devices Sensitized with Various Ru Complexes

Devices	CYC-B22	CYC-B23C	CYC-B23T	N719
SMM	0.9992	0.9954	0.9964	0.9900

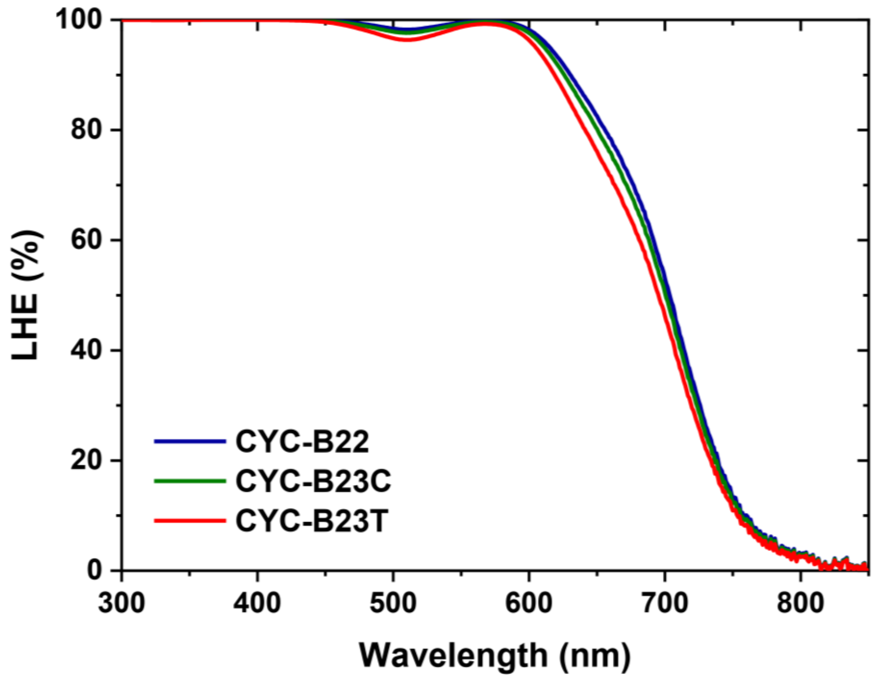

Figure S7. Light-harvesting efficiency (LHE) spectra of TiO_2 electrodes sensitized with **CYC-B22**, **CYC-B23C** and **CYC-B23T**, respectively.

Table S3. Electron Injection Efficiency (η_{inj}) of **CYC-B22**, **CYC-B23C**, **CYC-B23T** and **N719**

Complex	τ_{soln} (ns)[a]	τ_{film} (ns)	η_{inj} (%)[b]
**CYC-B22**	54.8	5.76	89.5
**CYC-B23C**	43.4	3.61	91.7
**CYC-B23T**	61.4	7.42	87.9
**N719**	105	9.53	90.9

[a] The solutions are degassed, and the excitation wavelength is 470 nm.
[b] The electron injection efficiency is calculated using the equation $\eta_{inj} = (1 - \frac{\tau_{film}}{\tau_{soln}}) \times 100\%$

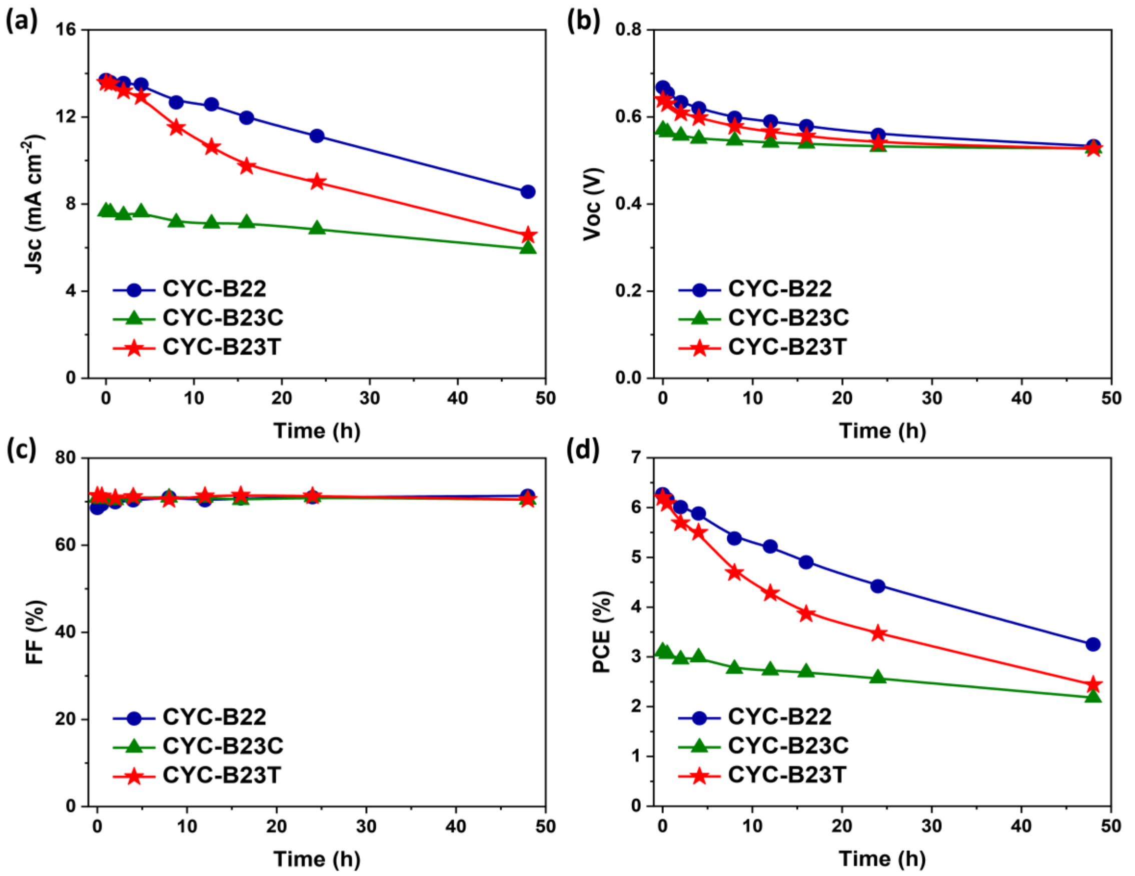

Figure S8. Evolution of (a) J_{sc}, (b) V_{oc}, (c) FF, and (d) PCE for devices sensitized with various Ru complexes under storage temperature of 70 ± 3 °C and measured under the standard testing conditions (STC).

3. Supporting References

(S1) Chen, C. Y.; Lu, H. C.; Wu, C. G.; Chen, J. G.; Ho, K. C. New Ruthenium Complexes Containing Oligoalkylthiophene-Substituted 1,10-Phenanthroline for Nanocrystalline Dye-Sensitized Solar Cells. *Adv. Funct. Mater.* **2007**, *17*, 29–36.

(S2) Klein, C.; Nazeeruddin, M. K.; Liska, P.; Censo, D. D.; Hirata, N.; Palomares, E.; Durrant, J. R.; Grätzel, M. Engineering of a Novel Ruthenium Sensitizer and Its Application in Dye-Sensitized Solar Cells for Conversion of Sunlight into Electricity. *Inorg. Chem.* **2005**, *44*, 178–180.

(S3) Mishra, A.; Pootrakulchote, N.; Fischer, M. K. R.; Klein, C.; Nazeeruddin, M. K.; Zakeeruddin, S. M.; Bäuerle, P.; Grätzel, M. Design and Synthesis of a Novel Anchoring Ligand for Highly Efficient Thin Film Dye-Sensitized Solar Cells. *Chem. Commun.* **2009**, 7146–7148.

(S4) Jang, S. R.; Yum, J. H.; Klein, C.; Kim, K. J.; Wagner, P.; Officer, D.; Grätzel, M.; Nazeeruddin, M. K. High Molar Extinction Coefficient Ruthenium Sensitizers for Thin Film Dye-Sensitized Solar Cells. *J. Phys. Chem. C* **2009**, *113*, 1998–2003.

(S5) Mishra, A.; Pootrakulchote, N.; Wang, M. K.; Moon, S. J.; Zakeeruddin, S. M.; Grätzel, M.; Bäuerle, P. A Thiophene-Based Anchoring Ligand and Its Heteroleptic Ru(II)-Complex for Efficient Thin-Film Dye-Sensitized Solar Cells. *Adv. Funct. Mater.* **2011**, *21*, 963–970.

(S6) Seo, J. Y.; Jeong, M. Y.; Seo, Y. N.; Lee, E. G.; Kim, Y. R.; Byoungchoo, P.; Kim, B. H.

Synthesis and Characterization of Novel Heteroleptic Ru(II) Bipyridine Complexes for Dye-Sensitized Solar Cell Applications. *Monatsh. Chem.* **2019**, *150*, 1445–1452.

(S7) Lee, J. H.; Seo, J. H.; Choi, Y. R.; Oh, H. J.; Huh, J. N.; Byoungchoo, P.; Tak, J.; Kim, B. H. Synthesis and Characterization of Heteroleptic Ru(II) Complexes Based on 4,4'-Bis((*E*)-styryl)-2,2'-Bipyridine as Ancillary Ligand and Application for Dye-Sensitized Solar Cells. *Helv. Chim. Acta* **2018**, *101*, e1800030.

(S8) Wu, S. J.; Chen, C. Y.; Chen, J. G.; Li, J. Y.; Tung, Y. L.; Ho, K. C.; Wu, C. G. An Efficient Light-Harvesting Ruthenium Dye for Solar Cell Application. *Dyes Pigm.* **2009**, *84*, 95–101.

(S9) Chen, C. Y.; Pootrakulchote, N.; Chen, M. Y.; Moehl, T.; Tsai, H. H.; Zakeeruddin, S. M.; Wu, C. G.; Grätzel, M. A New Heteroleptic Ruthenium Sensitizer for Transparent Dye-Sensitized Solar Cells. *Adv. Energy Mater.* **2012**, *2*, 1503–1509.
